# Nuclear receptor coactivator 6 (NCoA6) promotes cell proliferation, migration, and invasion in pancreatic cancer

**DOI:** 10.1002/cam4.6427

**Published:** 2023-08-08

**Authors:** Xin Wang, Yuming Jia, Xiaowu Xu, Yuheng Hu, Guixiong Fan, Desheng Jing, Zhilei Zhang, Chao Wang, Changfeng Song, Yi Qin, Li Peng

**Affiliations:** ^1^ Department of Emergency The Fourth Hospital of Hebei Medical University Shijiazhuang China; ^2^ Department of Hepatobiliary Surgery The Fourth Hospital of Hebei Medical University Shijiazhuang China; ^3^ Department of Pancreatic Surgery Fudan University Shanghai Cancer Center Shanghai China; ^4^ Department of Oncology, Shanghai Medical College Fudan University Shanghai China; ^5^ Shanghai Pancreatic Cancer Institute Shanghai China; ^6^ Pancreatic Cancer Institute, Fudan University Shanghai China

**Keywords:** NCoA6, pancreatic cancer, prognosis, proliferation, RNA‐sequencing

## Abstract

**Background:**

Nuclear receptor coactivator 6 (NCoA6) is overexpressed in various cancers and considered a multifunctional coactivator of various transcription factors and nuclear receptors. However, the role of NCoA6 in pancreatic ductal adenocarcinoma (PDAC) remains unclear.

**Methods:**

NCoA6 expression data in PDAC were extracted from TCGA and GTEx databases, and their correlation with survival outcomes were analyzed using the Kaplan–Meier plotter database. NCoA6 protein expression in PDAC tissues was evaluated using immunohistochemistry. RNA‐sequencing technology was used to sequence the transcriptome of NCoA6‐silenced PANC‐1 cells, followed by differential expression, GO/KEGG and GSEA analyses. The effects of NCoA6 on cell proliferation, migration, invasion, cell cycle, and apoptosis were determined in two representative cell lines (PANC‐1 and SW1990). Western blotting, qPCR, and co‐immunoprecipitation were performed to explore the mechanism of action of NCoA6 in PDAC cells.

**Results:**

NCoA6 expression was markedly increased in PDAC tissues, and high NCoA6 expression was associated with poor survival prognosis. However, there was no significant relationship between NCoA6 expression and metastasis in PDAC patients. Our RNA‐sequencing data analysis found 1194 significant differentially expressed genes between the control and NCoA6‐silenced PANC‐1 cells. GO/KEGG analysis results mainly focused on cytokine production, cytokine activity, and cytokine‐cytokine receptor interactions. GSEA results showed that the knockdown of NCoA6 affected the expression of histone deacetylase 1 (HDAC1) targeted genes. NCoA6 knockdown suppressed proliferation, migration, and invasion of PDAC cells. Finally, western blotting, qPCR, and co‐immunoprecipitation results showed that NCoA6 interacted with HDAC1 and that NCoA6 expression was negatively correlated with F‐box and WD repeat domain‐containing 7 (FBW7) and caudal‐related homeobox transcription factor 2 (CDX2) expression in pancreatic cancer.

**Conclusions:**

NCoA6 has a profound effect on cell proliferation, migration, invasion, and prognosis of PDAC and is potentially related to the expression of HDAC1, FBW7, and CDX2. Our results may provide novel therapeutic strategies for PDAC patients.

## 1 INTRODUCTION

According to GLOBOCAN 2020 estimates, pancreatic cancer leads to nearly 466,000 annual deaths, ranking as the seventh leading cause of cancer‐related death worldwide.^1^ Furthermore, the 5‐year survival rate of pancreatic cancer is still less than 10%,^2^ representing a rising public health burden worldwide. Pancreatic ductal adenocarcinoma (PDAC) accounts for nearly 95% of pancreatic cancer cases.^3^ Significant research progress has been made in recent years to efficiently understand the pathogenesis of PDAC and facilitate the optimization of existing therapeutic approaches, such as senescence‐induced vascular remodeling and pharmacological inhibition of KRAS^G12D^ in pancreatic cancer.^4^, ^5^ A better understanding of PDAC proliferation and metastasis is essential to improve clinical outcomes.

Nuclear receptor coactivator 6 (NCoA6), located on the 20th human chromosome, was first identified in breast cancer.^6^ NCoA6 is highly expressed in many cancers, including colorectal cancer, liver cancer, and melanoma.^7^, ^8^, ^9^ NCoA6, also known as a multifunctional coactivator of various transcription factors and nuclear receptors, modulates many critical cell functions.^10^ It was reported that NCoA6 promoted human placenta‐derived cell migration/invasion partially by activating NF‐κB‐mediated MMP9 transcription.^11^ However, the role of NCoA6 in PDAC and its underlying mechanism remain unclear.

Our team have been focused on the FBW7 gene (F‐box and WD repeat domain‐containing 7) in PDAC.^12^, ^13^, ^14^, ^15^ FBW7 is the substrate recognition component of the Skp1‐Cul1‐F‐box (SCF) ubiquitin ligase complex and plays a tumor‐suppressive role by degrading several critical oncoproteins, such as cyclin E and Notch. Our previous RNA‐sequencing results (GSE76443) indicated NCoA6 was a downregulated gene by overexpression of FBW7. Thus, we hypothesized that NCoA6 played an oncogenic role in PDAC progression and knockdown of NCoA6 could suppress cell proliferation, migration, and invasion potential by interacting with FBW7.

In this study, we performed a comprehensive investigation of NCoA6 role in PDAC based on public database, clinical tissues, cell culture, and RNA‐sequencing assays. Moreover, we further performed some preliminary exploration of its underlying mechanism using co‐immunoprecipitation analysis, qPCR, and western blotting. Our findings of NCoA6 could improve the understanding of molecular pathogenesis of PDAC and reveal novel prognostic and therapeutic targets for this cancer.

## 2 MATERIALS AND METHODS

### 2.1 Database and tissues

We downloaded mRNA expression data for The Cancer Genome Atlas (TCGA) pancreatic cancer samples and GTEx healthy pancreatic tissues from the UCSC Xena Database (http://xena.ucsc.edu/). All data profiles were normalized using log_2_(*x* + 1) transformation. The R “limma” package was used to perform differential expression analysis between the normal and tumor tissues. We then conducted survival analysis to assess the correlation between the mRNA expression of NCoA6 and survival profiles on the Kaplan–Meier plotter platform (www.kmplot.com). Receiver operating characteristic (ROC) curves were used to identify the diagnostic significance of NCoA6 in pancreatic cancer using the XIANTAO platform (www.xiantao.love). Furthermore, tumor (T) and adjacent normal tissues (ANT) were obtained from PDAC patients at Fudan University Shanghai Cancer Center (FUSCC). This study received informed consent from the patients and approval from the Institutional Research Ethics Committee of FUSCC. The clinical data and survival information of these patients were extracted from electronic medical records and telephone interviews.

### 2.2 Immunohistochemical (IHC) staining

IHC staining with antibodies against NCoA6 and FBW7 was conducted according to the standard procedures described in our previous article.^14^ In brief, the tissue slides were rehydrated using graded alcohols, submerged in citrate buffer, boiled for 2 min for antigen retrieval, blocked with 5% bovine serum albumin (BSA), and then incubated with antibodies. Incubation with primary antibodies for NCoA6 (1:100, Proteintech) and FBW7 (1:200, Bethyl) was at 4°C overnight, followed by polymeric HRP‐labeled anti‐rabbit IgG (Boster, China) at 37°C for 30 min. Tissue slides were scored to assess protein expression. Three fields of view were randomly selected for each slide and scored under an Eclipse Ci‐L microscope (Nikon, Japan). The levels of protein expression were calculated by multiplying the percentage of stained cells (0, 0%–10%; 1, 11%–25%; 2, 26%–50%; 3, 51%–75%; and 4, 76%–100%) by the staining intensity (0, no coloration; 1, pale yellow; 2, clay bank; and 3, brown). An overall immunohistochemical score >6 was considered high expression, whereas a score ≤6 was defined as low expression.

Seventy‐six PDAC patients with survival information were divided into high‐expression NCoA6 group (*n* = 58) and low‐expression NCoA6 groups (*n* = 18) based on immunohistochemistry scores to compare overall survival (OS) and disease‐free survival (DFS) outcomes by R package “survival”. Moreover, the R package “survivalROC” was used to calculate the time‐dependent ROC over 2 years.

### 2.3 Cell culture

We purchased PANC‐1 and SW1990 human pancreatic cancer cell lines from American Type Culture Collection. PANC‐1 cells were grown in Dulbecco's modified Eagle's medium with 10% fetal bovine serum (FBS), 100 U/mL penicillin, and 100 mg/mL streptomycin. The SW1990 cells were cultured in L‐15 medium with 10% FBS, 100 U/mL penicillin, and 100 mg/mL streptomycin. All cell cultures were kept in a humidified incubator at 37°C with 5% CO_2_.

### 2.4 Plasmids, short hairpin RNA (shRNA), and small interfering RNA (siRNA) treatments

To obtain NCoA6 silence plasmids, three shRNAs against NCoA6 were inserted into the pLKO.1‐TRC cloning vector (Addgene Plasmid 10878). To generate FBW7 overexpression plasmids, the coding sequences of human FBW7 or phospho‐deficient T205A FBW7 mutant (FBW7^T205A^, which was resistant to ERK activation and enhanced the stability of FBW7 protein in our previous study^13^) were cloned into the pCDH‐CMV‐MCS‐EF1‐puro lentiviral vector. Lentiviruses were produced in HEK293T cells by co‐transfecting plasmids with psPAX2 and pMD2.G vectors. Cells infected with NCoA6‐shRNAs and FBW7‐overexpressing vectors were selected using puromycin. The pLKO.1‐TRC cloning vector was used to silence HDAC1, and transfection of siRNA duplexes against HDAC1 was performed using Lipofectamine 2000 (Invitrogen). The shRNA and siRNA sequences were as follows:

NCoA6‐sh‐1: 5′‐GCAGATTATGACCAATCAAAT‐3′,

NCoA6‐sh‐2: 5′‐ACAAATGAACCCAGCTAATTT‐3′,

NCoA6‐sh‐3: 5′‐GCCCATTGTTGGTCAACTTAT‐3′,

HDAC1‐si‐1: 5′‐GCGACTGTTTGAGAACCTT‐3′,

HDAC1‐si‐2: 5′‐GGGATCGGTTAGGTTGCTT‐3′,

HDAC1‐si‐3: 5′‐AGGCGGTGGTTACACCATT‐3′.

### 2.5 Protein extraction and western blotting

Protein extraction and western blotting were performed as described in our previous research.^16^ Antibodies against NCoA6 (1:1000), E‐cadherin (1:5000), N‐cadherin (1:3000), FBW7 (1:1000), CDX2 (1:1000), cyclin‐dependent kinase 4 (CDK4, 1:2000), cyclin‐dependent kinase 2 (CDK2, 1:5000), Cyclin D1 (1:5000), Cyclin E1 (1:1000), and Cyclin A2 (1:2000) were obtained from Proteintech. Antibodies against p21 and p27 (1:1000) were purchased from Cell Signaling Technology (Danvers, MA, USA). Antibodies against HDAC1, Vimentin, and AcH4 (1:1000) were purchased from Abcam. The AcH3 antibodies (1:1000) and β‐actin (1:10000) were produced by Abclonal. All secondary antibodies were purchased from Abclonal. Trichostatin A (TSA), an HDAC1 inhibitor, was purchased from MedChem Express.

### 2.6 RNA isolation and quantitative real‐time PCR

Total RNA isolation and quantitative real‐time PCR were conducted as described previously.^16^ Relative mRNA expression for each gene was quantified relative β‐actin for three times using the ΔΔCt method. Primers used were as follows:

NCoA6:

5′‐ACCGTTGCCTGGAGAACAAGGA‐3′ (forward),

5′‐GAGTTGAGGAGGCATCTGCTGA‐3′ (reverse).

CDX2:

5′‐GCTGGAGCTGGAGAAGGAGTTTC‐3′ (forward),

5′‐AAGGGCTCTGGGACACTTCTCAGA‐3′ (reverse).

β‐actin:

5′‐TCCTTCCTGGGCATGGAGT‐3′ (forward),

5′‐CAGGAGGAGCAATGATCTTGAT‐3′ (reverse).

### 2.7 RNA‐sequencing and data analysis for NCoA6‐NC/sh PANC‐1 cells

PANC‐1 cells were extracted using TRIzol/chloroform and purified using poly‐T oligo‐attached magnetic beads. RNA quality was assessed using an Agilent Bioanalyzer 2100 system. A total amount of 3 μg RNA per sample was used as input material for the RNA sample preparations. The library for RNA‐Seq processing was constructed according to the NEBNext® Ultra™ RNA Library Prep Kit for Illumina® (NEB, USA) and previous research.^17^ After second‐strand cDNA synthesis, the remaining overhangs were converted into blunt ends via exonuclease/polymerase activity. Library quality was assessed using the Agilent Bioanalyzer 2100 system. After cluster generation, the library preparations were sequenced on an Illumina NovaSeq 6000 system and 150 bp paired‐end reads were generated. After quality control and cleaned read mapping to the reference genome, FeatureCounts v1.5.0‐p3 was used to count the read numbers mapped to each gene. Differential expression analysis of the two groups was performed using the “DESeq2” R package (version 1.16.1). The threshold of differentially expressed genes (DEGs) was |log2 FC|>1 and *p* < 0.05. Then, the volcano plot and heatmap were visualized using the “ggplot2” R package. Gene Ontology/Kyoto Encyclopedia of Genes and Genomes (GO/KEGG) analysis was performed to determine the potential mechanism and downstream signaling pathways of NCoA6. Gene set enrichment analysis (GSEA) was conducted using the Hallmark gene sets from the Molecular Signatures Database (MSigDB).^18^

### 2.8 Cell proliferation assay

Cell proliferation was evaluated using the CCK8 assay. Briefly, PDAC cells were plated in 96‐well plates (2 × 10^3^ cells/well, maintained in 200 μL of medium) and cultured for the indicated times, then incubated with CCK8 reagents for an additional 2 h at 37°C. Absorbance was measured at 450 nm using a microplate reader.

### 2.9 Colony formation assay

Cells were seeded in 6‐well plates at 500 cells/well and cultured for 2 weeks. Colonies were fixed using 4% paraformaldehyde, stained with 0.1% crystal violet, and counted under an inverted microscope. The number of colonies was calculated as the average of three independent experiments.

### 2.10 Cell cycle assessment

Cells were collected and fixed with 70% alcohol at 4°C overnight. Propidium iodide, a staining buffer containing RNase, was added to the cells and then incubated at 37°C for 30 min in the dark. The cell cycle distribution was measured using a BD FACSCalibur flow cytometer. MultiCycle software (Beckman Coulter) was used for data analysis.

### 2.11 Cell apoptosis analysis

Cells were subjected to fluorescein isothiocyanate‐conjugated Annexin V‐EGFP and PI (CellorLab) for 10 min in the dark and then analyzed by flow cytometry to determine the cell apoptosis rate.

### 2.12 Scratch assay

Cells were plated in 6‐well plates and cultured overnight in serum‐free medium. Scratches were created using a 10 μL pipette tip. The plates were washed and replaced with fresh serum‐free medium at 0, 12, and 24 h after scratching. Scratches were recorded and measured using an inverted microscope.

### 2.13 Transwell migration and invasion assays

Cell migration and invasion assays were performed using a 24‐transwell chamber (8‐um pore) with or without Matrigel. Cells (6 × 10^4^) were seeded in the upper chamber of serum‐free medium. Medium (600 μL) with 10% FBS was added into the lower chamber. After incubation for 24 h, non‐migrating or non‐invading cells were removed and the cells penetrating the membrane were stained and counted under a microscope in five randomly selected fields.

### 2.14 Co‐immunoprecipitation

Cells were washed in PBS and subjected to cold WB and IP lysis buffer containing 1 mM PMSF. Lysates were centrifuged at 14,000 g and 4°C for 10 min. The supernatant was pre‐purified using rabbit IgG (Beyotime) and pretreated with protein A/G magnetic beads (Beyotime). The samples were incubated with 4 μg NCoA6 antibody (Proteintech) with rotating at 4°C overnight. Protein A/G magnetic beads were added and incubated for 3 h. The precipitate was collected and loaded onto SDS‐PAGE gel for western blotting.

### 2.15 Statistics

Data are presented as the mean ± SD unless otherwise stated. All statistical analyses were conducted using SPSS software (IBM, version 26.0), GraphPad Prism 8, or RStudio (PBC, version 1.4.1717). Time‐dependent ROC curves were used to assess the predictive value of NCoA6 expression in PDAC tissues for overall and DFS at different time points. Two‐tailed unpaired Student's *t*‐test, chi‐squared test, log‐rank test, and Cox regression were used to analyze the data. After univariate Cox regression analysis, we selected significant factors for multivariate Cox regression analysis. Differences with *p < 0.05, **p < 0.01, and ***p < 0.001 were considered significant.

## 3 RESULTS

### 3.1 Expression of NCoA6 was negatively associated with PDAC prognosis

**FIGURE 1:**
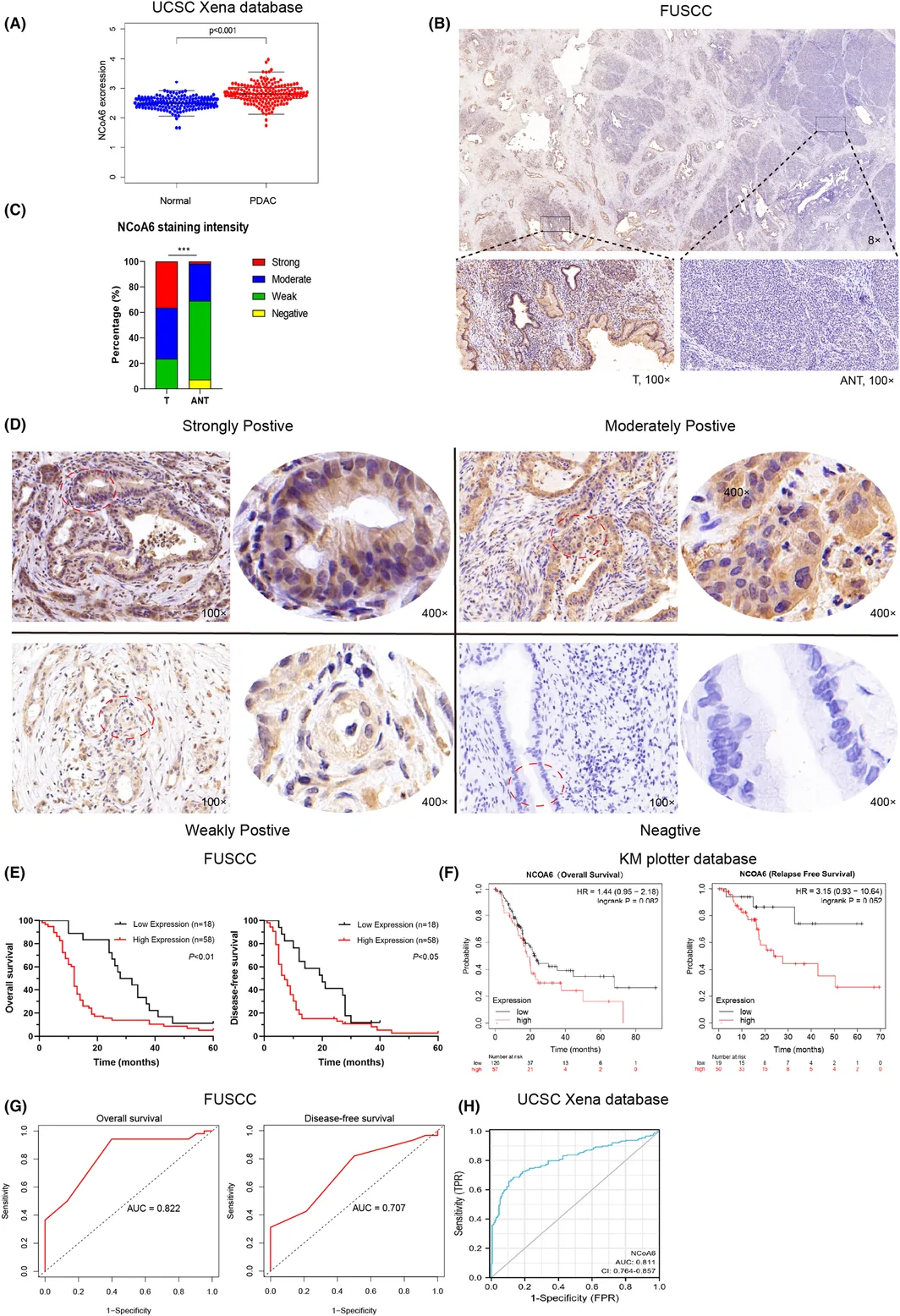
Upregulation of NCoA6 expression is correlated with PDAC progression. (A) NCoA6 expression is significantly increased in PDAC samples compared with normal tissues from TCGA and GTEx. (B) Representative images of IHC staining for NCoA6 in PDAC tumor tissues (T) and adjacent normal tissues (ANT). (C) IHC score of NCoA6 expression in PDAC and ANT (*n* = 55, *p* < 0.001). (D) Representative images of IHC staining for NCoA6 in tissue microarrays. (E) Kaplan–Meier analysis of NCoA6 expression and overall survival (*p* < 0.01) and disease‐free survival (*p* < 0.05) of patients with PDAC from FUSCC. (F) The high NCoA6 expression group is associated with worse overall and disease‐free survival according to the Kaplan–Meier plotter database (hazard ratio [HR] = 1.44 and 3.15, respectively). (G) Time‐dependent ROC curve shows that NCoA6 expression in PDAC tissues from FUSCC is related to the 2‐year overall and disease‐free survival of patients (AUC = 0.822 and 0.707, respectively). (H) The ROC analysis from UCSC shows the moderate prognostic value of NCoA6 expression level in PDAC tissues (AUC = 0.811, *p* < 0.001).

Pancreatic cancer sequencing data from TCGA and GTEx showed that NCoA6 expression was upregulated in 178 PAAD samples compared to 171 normal tissues (Figure 1A). The IHC staining results of clinical samples further confirmed the upregulation of NCoA6 in cancer tissues compared to ANTs (*n* = 55) (Figure 1B,C). Next, we explored the relationship between clinicopathological features and NCoA6 expression in 76 patients with PDAC. Figure 1D shows the representative images of IHC staining for NCoA6 in tissue microarrays. The high IHC score for NCoA6 correlated with tumor size and TNM stage (Table 1). Cox regression analysis revealed that NCoA6 is an independent prognostic marker of OS and DFS outcomes for PDAC (Table 2). Additionally, Kaplan–Meier analysis of FUSCC data (Figure 1E) and the public K–M Plotter database (Figure 1F) revealed that high expression of NCoA6 correlated with poor survival in PDAC cases. Finally, time‐dependent ROC (Figure 1G) and ROC analysis (Figure 1H) showed that the prognostic value of NCoA6 expression in PDAC tissues was moderate.

**TABLE 1:** Relationship between clinicopathological features and NCoA6 expression in patients with pancreatic cancer.

Variables	NCoA6 expression		*p* value
	Low (*n* = 18)	High (*n* = 58)	
Gender			0.542
Male	12 (66.7%)	34 (58.6%)	
Female	6 (33.3%)	24 (41.4%)	
Age (years)			0.724
>60	12 (66.7%)	36 (62.1%)	
≤60	6 (33.3%)	22 (37.9%)	
Tumor size (cm)			0.007^**^
>4	2 (11.1%)	27(46.6%)	
≤4	16 (88.9%)	31 (53.4%)	
Tumor differentiation			0.245
Well/Moderately	13 (72.2%)	33 (56.9%)	
Poorly	5 (27.8%)	25 (43.1%)	
Tumor thrombi			1.000
Positive	4 (22.2%)	11 (19.0%)	
Negative	14 (77.8%)	47 (81.0%)	
Perineural invasion			0.220
Positive	18 (100.0%)	50 (86.2%)	
Negative	0 (0%)	8 (13.8%)	
Lymph node metastasis			0.215
Positive	6 (33.3%)	29 (50.0%)	
Negative	12 (66.7%)	29 (50.0%)	
Distant metastasis			1.000
Positive	1 (5.6%)	3(5.2%)	
Negative	17 (94.4%)	55(94.8%)	
TNM stage			0.009^**^
I + II	17 (94.4%)	36 (62.1%)	
III + IV	1 (5.6%)	22 (37.9%)	

*p < 0.05, **p < 0.01, and ***p < 0.001 are considered significant.

**TABLE 2:** Univariate and multivariate Cox regression of overall survival and disease‐free survival of patients with PDAC.

Variable		Overall survival				Disease‐free survival			
		Univariate		Multivariate		Univariate		Multivariate	
		HR (95% CI)	*p* value	HR (95% CI)	*p* value	HR (95% CI)	*p* value	HR (95% CI)	*p* value
Gender	Male	0.732 (0.454–1.180)	0.201			0.816 (0.504–1.319)	0.406		
Age	>60	0.970 (0.598–1.572)	0.900			0.858 (0.523–1.408)	0.545		
Tumor size	>4	1.787 (1.105–2.889)	0.018^*^	1.381 (0.826–2.307)	0.218	1.450 (0.897–2.345)	0.130		
Tumor differentiation	Poorly	1.790 (1.109–2.889)	0.017^*^	1.553 (0.926–2.603)	0.095	1.669 (1.032–2.700)	0.037^*^	1.608 (0.973–2.659)	0.064
Tumor thrombi	Positive	1.176 (0.654–2.115)	0.589			0.904 (0.502–1.630)	0.737		
Perineural invasion	Positive	0.580 (0.271–1.241)	0.160			0.661 (0.311–1.405)	0.282		
Lymph node metastasis	Positive	2.040 (1.267–3.284)	0.003^**^	1.677 (1.015–2.773)	0.044^*^	1.659 (1.035–2.658)	0.035^*^	1.426 (0.872–2.334)	0.158
TNM stage	III + IV	1.257 (0.758–2.084)	0.376			1.428 (0.860–2.370)	0.168		
NCoA6 expression	High	2.201 (1.245–3.890)	0.007^**^	1.966 (1.079–3.584)	0.027^*^	2.008 (1.136–3.548)	0.016^*^	2.040 (1.145–3.632)	0.015^*^

*p < 0.05, **p < 0.01, and ***p < 0.001 are considered significant.

### 3.2 RNA‐Seq data analysis

**FIGURE 2:**
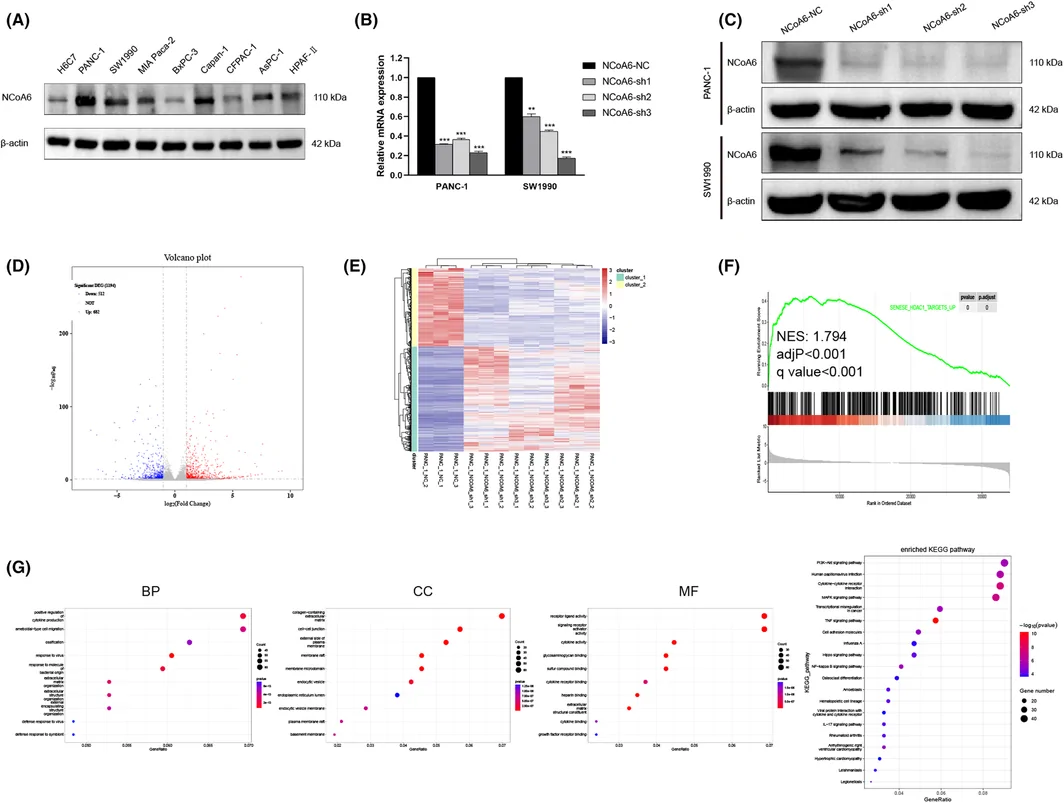
GO/KEGG analysis and GSEA of our RNA‐sequencing data. (A) Western blotting analysis of NCoA6 in PDAC lines. The H6C7 cell line was included as a negative control for the detection of endogenous NCoA6 expression, and β‐Actin was used as a control. (B) RT‐qPCR and (C) western blotting analysis of NCoA6 expression in NCoA6 PANC‐1 and SW1900 cells. The volcano plot (D) and heatmap (E) of gene alterations for NCoA6‐NC/sh PANC‐1 cells. (F) GSEA shows that SENESE_HDAC1_TARGETS_UP, the gene set from Human MSigDB Collections, is significantly enriched in the RNA‐sequencing dataset of NCoA6‐NC/sh PANC‐1 cells. (G) The main enriched GO/KEGG results of NCoA6‐NC/sh PANC‐1 cells.

In vitro, NCoA6 expression levels in eight human pancreatic cancer cell lines were much higher than those in normal human pancreatic ductal epithelium H6C7 cells (Figure 2A). We used three different shRNA constructs in PANC‐1 and SW1990 cells to stably silence NCoA6 expression. The silencing effect of each shRNA was determined using RT‐qPCR and western blotting (Figure 2B,C). A total of 1194 significant DEGs between the control and NCoA6‐silenced PANC‐1 cells were obtained using RNA‐Seq data analysis, including 682 upregulated, and 512 downregulated genes. The differences between the two groups could be distinguished in volcano plots and heatmaps (Figure 2D,E). The DEGs significantly dysregulated in the RNA‐Seq dataset are shown in Tables S1 and S2. Our GO analysis results showed that variations in the biological processes (BP) of DEGs were mainly enriched in the positive regulation of cytokine production, ameboidal cell migration, and response to virus (Figure 2G). The variations in cell component (CC) were notably enriched in the collagen‐containing extracellular matrix, cell–cell junction, and external side of plasma (Figure 2G). Variations in the molecular function (MF) of DEGs were significantly enriched in signaling receptor activity, cytokine activity, and growth factor receptor binding (Figure 2G). KEGG pathway analysis revealed that all DEGs were primarily enriched in the PI3K‐Akt signaling pathway, cytokine‐cytokine receptor interaction, and MAPK signaling pathway (Figure 2G). Furthermore, GSEA showed that SENESE_HDAC1_TARGETS_UP (genes up‐regulated in U2OS cells upon knockdown of HDAC1 by RNAi, http://www.gsea‐msigdb.org/gsea/msigdb/human/geneset/SENESE_HDAC1_TARGETS_UP.html?keywords=HDAC1), the gene set from Human MSigDB Collections, was significantly enriched in the RNA‐Seq dataset of NCoA6‐NC/sh PANC‐1 cells (Figure 2F). The statistical values of the GO, KEGG, and GSEA analyses are shown in Tables [Link], [Link].

### 3.3 NCoA6 promoted the proliferation and migration/invasion of PDAC

**FIGURE 3:**
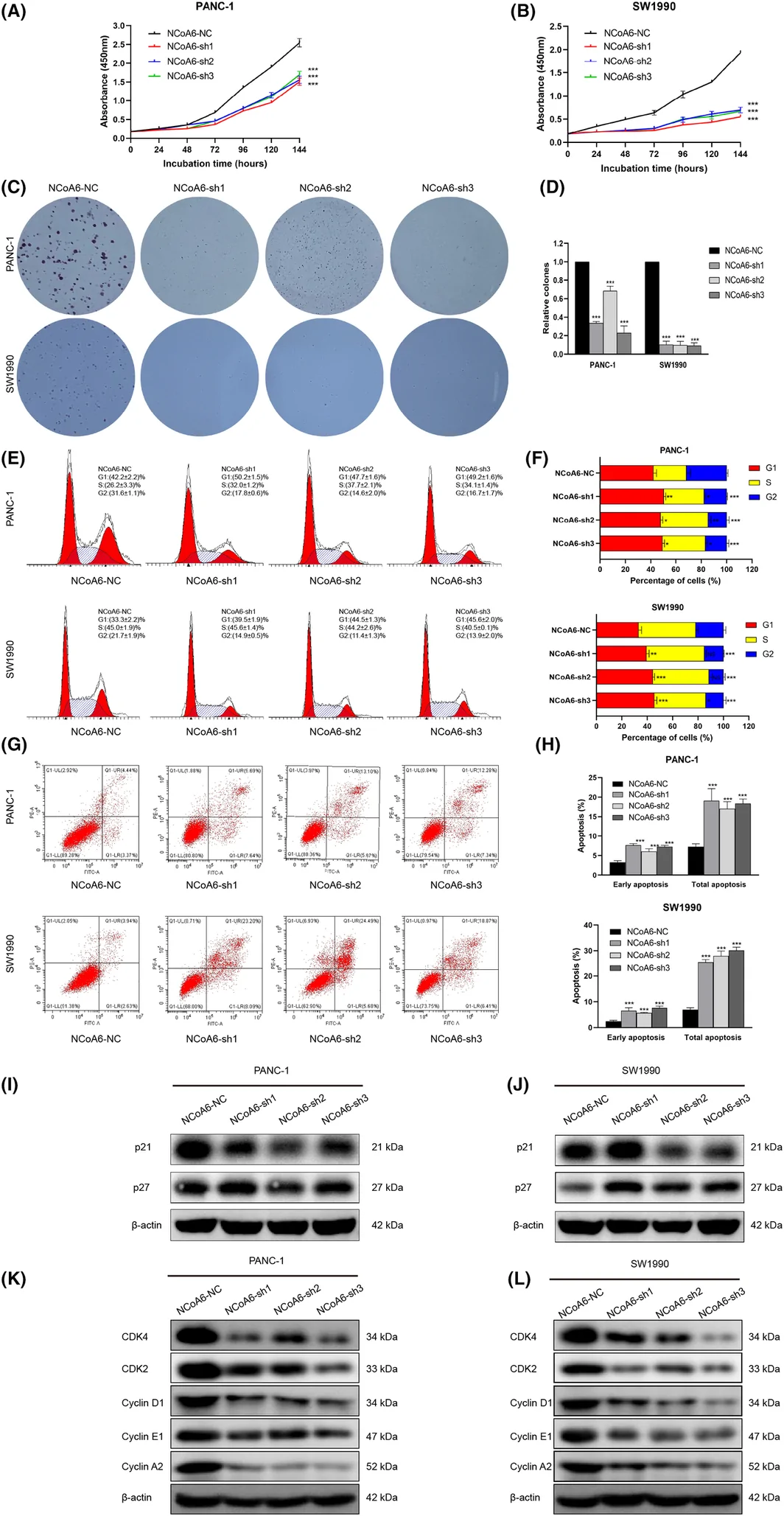
NCoA6 knockdown decreases the proliferation, cell cycle, and apoptosis of pancreatic cancer cells. CCK‐8 assays (A, B) and colony formation assays (C, D) were used to test the proliferation of PDAC cells transfected with NCoA6 shRNAs. The representative images and statistical results of the cell cycle (E, F) and apoptosis (G, H) using flow cytometry. (I, J) Western blotting analysis of p21 and p27 expression in NCoA6‐silenced PANC‐1 and SW1990 cells. (K, L) Western blotting analysis of CDK4, CDK2, Cyclin D1, Cyclin E1, and Cyclin A2 expression in NCoA6‐silenced PANC‐1 and SW1990 cells.

**FIGURE 4:**
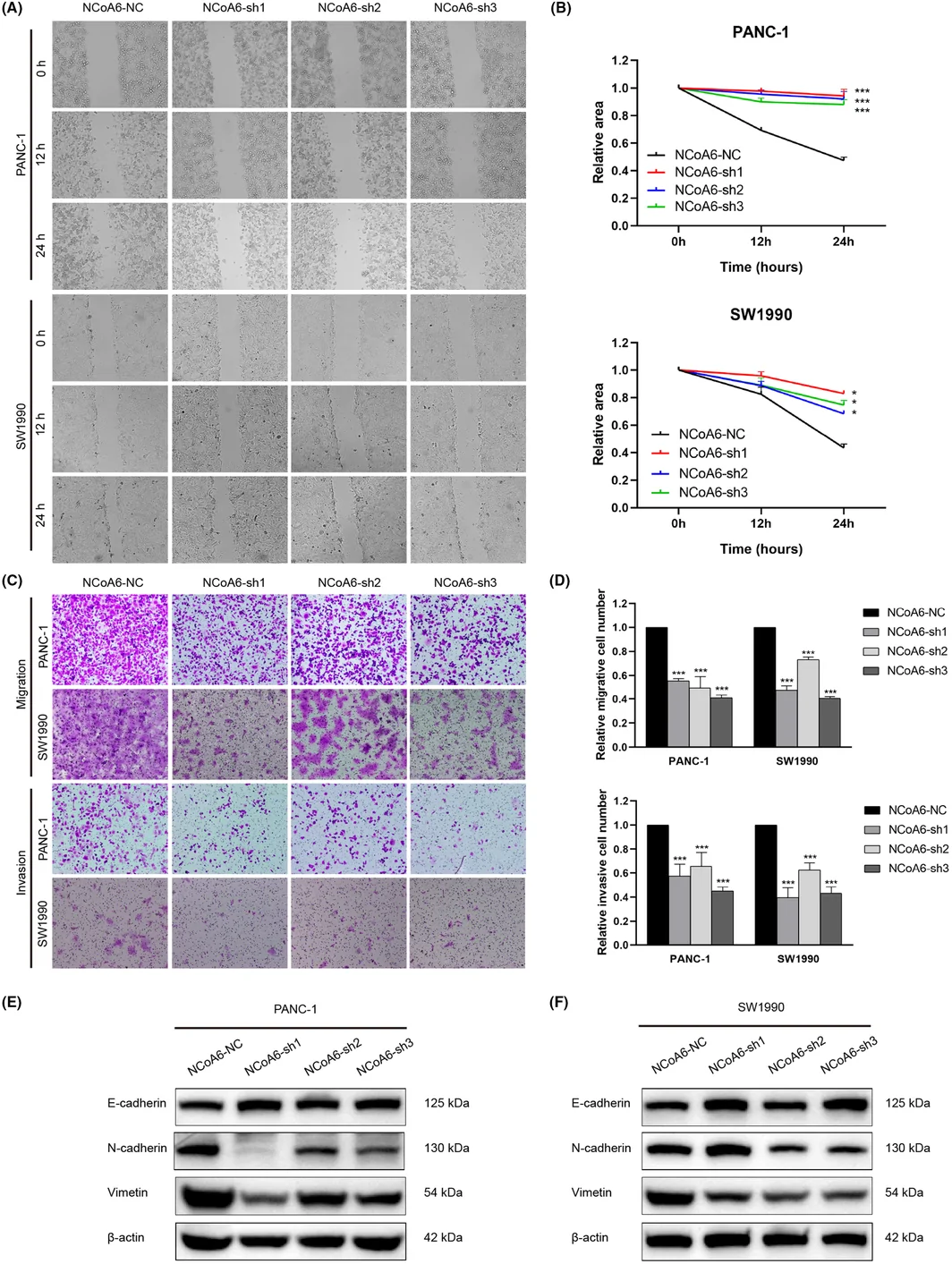
NCoA6 knockdown decreases the migration and invasion capacity of pancreatic cancer cells. Scratch assays (A, B) and transwell migration and invasion assays (C, D) were performed to assess the effect of NCoA6 silencing on pancreatic cancer cell lines. (E, F) Western blotting analysis of EMT protein expression in NCoA6‐silenced PANC‐1 and SW1990 cells.

Various functional assays were performed on the silenced cells to investigate the role of NCoA6 in PDAC malignancy. CCK8 assays, colony formation assays, and flow cytometry revealed that NCoA6 knockdown in PANC‐1 and SW1990 cells reduced cell growth and promoted cell cycle arrest and apoptosis (Figure 3A–H). Accordingly, we detected upregulation of the cell cycle inhibitor protein p27 and downregulation of CDK4, CDK2, cyclin D1, cyclin E1, and cyclin A2 in NCoA6‐silenced cells (Figure 3I–L). In addition, we evaluated changes in invasiveness and epithelial to mesenchymal transition (EMT)‐related proteins. Our results showed that when NCoA6 was knocked down, migration and invasion abilities were significantly weakened (Figure 4A–D), which correlated with changes in the expression of E‐cadherin, N‐cadherin, and Vimentin (Figure 4E,F). These data indicate that NCoA6 expression increased during PDAC progression and promoted the growth, migratory, and invasive abilities of PDAC cells.

### 3.4 NCoA6 interacted with HDAC1 and negatively correlated with FBW7 and CDX2 expression in PANC‐1 and SW1990 cells

**FIGURE 5:**
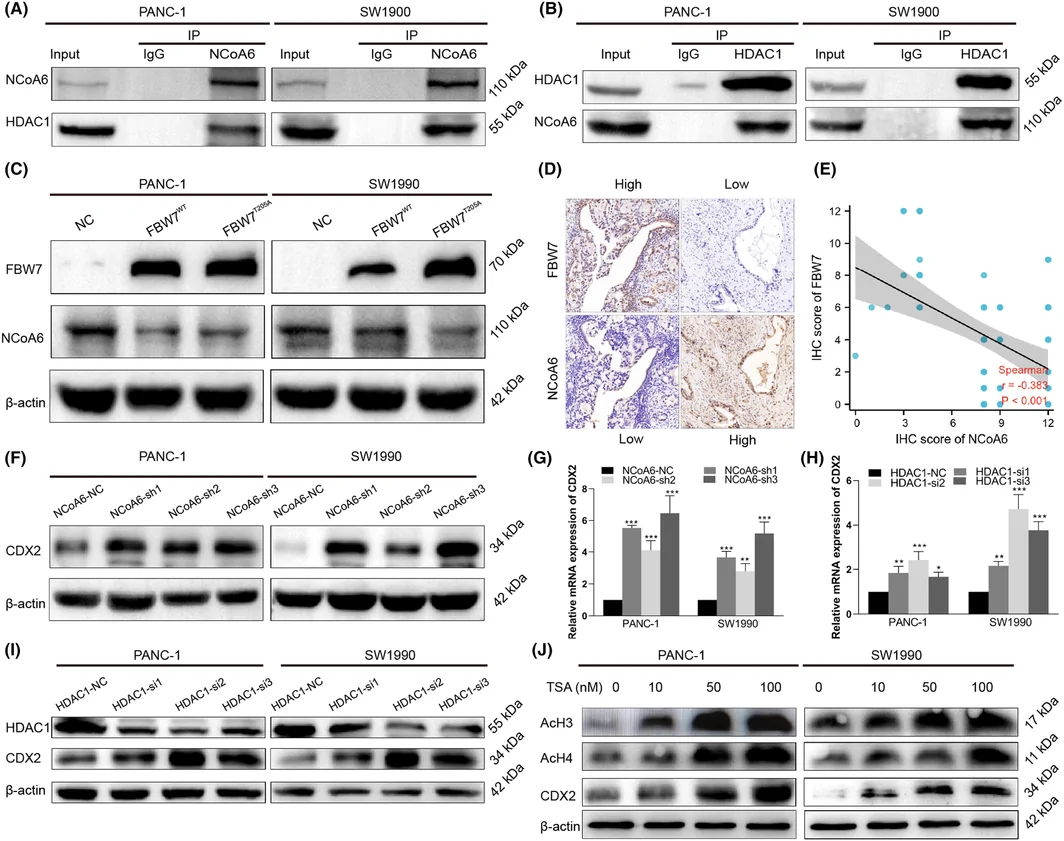
Potential endogenous interactions of NCoA6, HDAC1, FBW7, and CDX2. (A, B) Co‐immunoprecipitation validated NCoA6 interaction with HDAC1 in PDAC cells. NCoA6 expression was negatively correlated with FBW7 and CDX2 expression. (C) Western blotting validated NCoA6 downregulation in FBW7 overexpressing PANC‐1 and SW1990 cells. (D, E) Correlation analysis of NCoA6 expression and FBW7 expression in PDAC tissues, as determined by the IHC score (***p < 0.001). (F, G) Western blotting and RT‐qPCR validated CDX2 upregulation in NCoA6 knockdown PDAC cells. (H, I) Western blotting and RT‐qPCR validated CDX2 upregulation in HDAC1 knockdown PDAC cells. (J) AcH3, AcH4, and CDX2 were upregulated in HDAC inhibitor TSA‐treated PDAC cells (western blotting).

**FIGURE 6:**
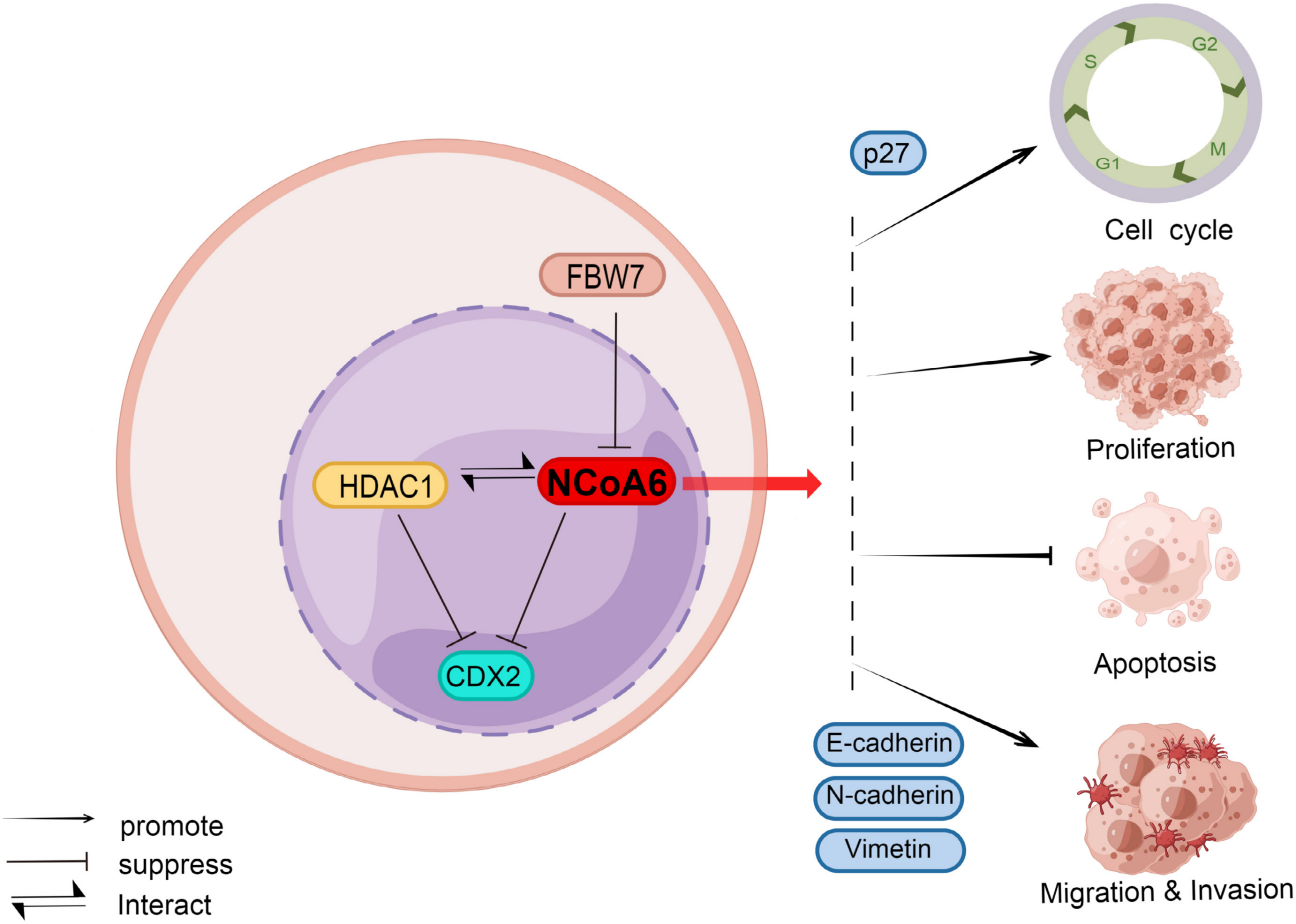
Schematic representation of the working model designed using FigDraw. NCoA6 plays an important role in promoting PDAC cell proliferation, migration, invasion and progression, and is potentially related to the expression of HDAC1, FBW7, and CDX2.

Several experiments were performed to explore the possible mechanisms of action of NCoA6 in PDAC. Co‐immunoprecipitation results validated the interaction between NCoA6 and HDAC1 in PDAC cell lines (Figure 5A,B). We screened our previous whole‐genome expression microarray (GEO accession number: GSE76443) and found that NCoA6 expression was downregulated by FBW7. Western blotting results showed that FBW7 overexpression in PANC‐1 and SW1990 cells significantly downregulated the expression of NCoA6 (Figure 5C). IHC of PDAC specimens also supported the negative correlation between FBW7 and NCoA6 expression (Figure 5D,E). Our RNA‐Seq data analysis showed that CDX2 was upregulated by a lack of NCoA6 expression (log_2_FC = 2.43, *p* < 0.001; Table S1). Interestingly, western blotting and RT‐qPCR validated that the separate silencing of NCoA6 and HDAC1 by si/shRNAs contributed to the significant upregulation of CDX2 in PANC‐1 and SW1990 cells (Figure 5F–I). The HDAC1 inhibitor (TSA) also promoted CDX2 expression in PDAC cell lines (Figure 5J). Taken together, these results suggest that NCoA6 interacts with HDAC1 and negatively correlates with FBW7 and CDX2 expression in PDAC cells (Figure 6).

## 4 DISCUSSION

In this in vitro study, we found that NCoA6 was highly expressed in PDAC cells and tissues and that high expression of NCoA6 was closely related to poor survival of patients with PDAC. NCoA6 knockdown remarkably suppressed cell proliferation and migration/invasion and promoted cell cycle arrest in PDAC cells. These changes induced by NCoA6 knockdown may be associated with HDAC1, FBW7, and CDX2 expression.

To our knowledge, this is the first study to demonstrate the significance of NCoA6 expression in PDAC. NCoA6 is often overexpressed in various cancer types, and its overexpression levels are associated with poor prognoses for cancer patients. For example, NCoA6 overexpression has been demonstrated in liver cancer compared to ANTs and is closely correlated with poor prognosis.^9^ Consistent with our hypothesis, this study found that NCoA6 was highly expressed in PDAC cell lines and tissues and that high expression of NCoA6 was a risk factor for poor prognosis in patients with PDAC. These results suggest that aberrant NCoA6 expression plays an oncogenic role in PDAC.

To further verify this hypothesis, several cell experiments, including CCK8, colony formation, scratch, and transwell assays, were performed. Our data showed that NCoA6 knockdown in PDAC cells markedly suppressed cell proliferation, migration, and invasion. These results are consistent with those of previous studies,^9^ highlighting the crucial role of NCoA6 in cancer development. However, our data could not exclude the possibility that the loss of cell viability could affect the results of migration/invasion induced by NCoA6 knockdown. In human placenta‐derived cells, NCoA6 knockdown significantly attenuated cell invasion/migration but had no significant effect on proliferation and viability.^11^ Thus, we speculated that the inhibitory effect of NCoA6 on cell proliferation and viability may differ in various cell types.

One of the main characteristics of cancer is malignant cell transformation, which results from disrupted cell‐cycle regulation and continuous uncontrolled growth. Ample evidence has indicated that abnormal cyclin proteins and apoptosis are closely associated with cell proliferation and malignancy.^19^ To detect the effect of NCoA6 on the cell cycle and apoptosis, we performed various experiments, which showed that the number of apoptotic cells and p27 expression were much higher in the NCoA6 knockdown group than in the control group. As p27 is a cyclin–CDK inhibitor, we further detected the expression of cyclins and CDKs that showed different degrees of decline. KEGG analysis also verified that NCoA6 was involved in the regulation of the PI3K‐Akt and MAPK signaling pathways, which have been reported to affect autophagy and apoptosis.^20^ Therefore, we speculated that NCoA6 knockdown might arrest the cancer cell cycle, inhibit cell proliferation, and ultimately promote apoptosis. Several studies have confirmed that NCoA6 deletion can lead to early embryonic death or slow growth in mice, potentially by disrupting the cell cycle and increasing apoptosis.^10^, ^21^, ^22^, ^23^ Qi et al. found that NCoA6‐deficient mice showed decreased ductal branch number, gland density, and milk secretion, which were related to increased apoptosis in the terminal end buds of the breast.^24^ These findings suggest that NCoA6 is an important anti‐apoptotic gene that regulates cell growth and survival.

EMT has long been implicated as a crucial molecular mechanism in cancer metastasis.^25^ Thus, we validated the functional role of NCoA6 in the migration and invasion of PDAC cells by measuring the expression of EMT markers after gene silencing. In our study, E‐cadherin expression increased in NCoA6 knockdown cells, whereas N‐cadherin and Vimentin expression decreased. Importantly, our GO analysis showed that genes altered following NCoA6 knockdown were significantly enriched in extracellular matrix and cytokine activity. It has been reported that various chemokines and cytokines secreted by cancer‐associated fibroblasts contribute to EMT progression in PDAC cells.^26^ Our study showed that there was no statistic difference of the status of lymph node metastasis and distant metastasis between high and low NCoA6 expression groups, which may be due to the small number of patients that already had metastasis at the time of diagnosis. Meanwhile, high NCoA6 expression closely correlated with poor prognosis, including OS and DFS. Further experimental work will be needed to confirm the role NCoA6 in PDAC metastasis.

Accumulating evidence has shown that FBW7 serves as a tumor suppressor by regulating the phosphorylation‐dependent ubiquitination and proteasomal degradation of oncoproteins.^27^, ^28^ Our previous study found that ERK kinase causes the phosphorylation and destabilization of FBW7, thus regulating pancreatic cancer cell proliferation and tumorigenesis.^12^ Considering the important role of FBW7 in PDAC development, we reanalyzed our previous high‐throughput gene expression profiling array (GEO accession number GSE76443), which showed that NCoA6 was downregulated by FBW7. Western blotting and IHC of PDAC samples verified that NCoA6 expression was highly negatively correlated with FBW7 expression. It has been reported that the inactivation of FBW7 significantly affects lipid metabolism, proliferation, and survival of cancer cells, potentially by activating the PI3K‐AKT signaling axis.^29^ Interestingly, our KEGG analysis also showed that NCoA6 was associated with the PI3K‐AKT and MAPK signaling pathways. These results demonstrate that FBW7 affects the expression of NCoA6. However, a limitation of this study is that we did not expound how FBW7 regulates NCoA6. Previous research reported that binding sites for H4K12ac were observed within developmentally important promoters of NCoA6.^30^ We hypothesized that FBW7 regulates NCoA6 expression through ubiquitination or acetylation of a transcription factor for NCoA6 transcription. However, this hypothesis requires further experimental validation.

HDAC1 is a subunit of multiprotein nuclear complexes that plays a crucial role in transcriptional repression and epigenetic landscaping.^31^ HDAC1 knockout is embryonically lethal, indicating its vital role in cell proliferation, apoptosis, and morphogenesis.^32^ In breast cancer cells and osteosarcoma, HDAC1 knockdown causes cell cycle arrest and cell growth inhibition and increases the proportion of apoptotic cells,^33^ while HDAC1 overexpression promotes prostate cancer cell proliferation.^34^ Furthermore, HDAC1 overexpression was found to be associated with advanced tumor stage and poor survival in gastric and colorectal cancers.^35^, ^36^ To investigate the relationship between NCoA6 and HDAC1, we performed GSEA of the RNA‐Seq data to identify key molecules and downstream signaling pathways. Interestingly, GSEA results using hallmark gene sets also showed that the knockdown of NCoA6 induced alterations in many HDAC1‐mediated genes. Furthermore, we verified, by co‐immunoprecipitation, that strong molecular interactions exist between NCoA6 and HDAC1.

CDX2 is a member of the homeobox gene family and plays an important role in the proliferation and differentiation of intestinal epithelial cells.^37^, ^38^ Tumor suppressor gene CDX2 regulated various physiological processes, such as tumorigenesis.^39^ Overexpression of CDX2 is considered a reliable prognostic factor and sensitive biomarker for gastrointestinal tumors.^40^ In our study, CDX2 expression was much higher in NCoA6‐knockdown cells than in control cells, as confirmed by RNA‐Seq data analysis, RT‐qPCR, and western blotting. Furthermore, CDX2 expression was upregulated by HDAC1 knockdown and TSA treatment. These results indicate that NCoA6 interacts with HDAC1 and NCoA6 is negatively correlated with FBW7 and CDX2. However, further investigation is needed to explore this complicated gene–gene interaction network.

Our study had several limitations. First, all experiments were performed in PDAC cells and tissues in vitro. Further studies using animal models and prospective clinical data are required to validate the role of NCoA6 in patients with PDAC. Second, the causal relationship between the anti‐tumor effects of NCoA6 knockdown and the molecular changes observed in this study warrants further investigation. Third, we found that NCoA6 knockdown decreased EMT marker expression in vitro and that NCoA6 overexpression was associated with late TNM stage. However, data on tumor metastasis should be further examined in an experimental metastasis mouse model by injecting NCoA6‐sh PDAC cells.

## 5 CONCLUSIONS

In summary, we demonstrated that NCoA6 is an oncogene involved in the progression of pancreatic cancer. Based on sequencing data from TCGA, clinical PDAC samples, and in vitro experiments, increased NCoA6 expression was linked to poor outcomes in patients with PDAC, potentially by facilitating the proliferation and metastatic ability of PDAC cells. Mechanistically, NCoA6 interacted with HDAC1 and was negatively correlated with the expression of FBW7 and CDX2. Therefore, targeting NCoA6 to inhibit tumor malignancy may be a novel therapeutic approach for PDAC.

## AUTHOR CONTRIBUTIONS

**Xin Wang:** Data curation (lead); formal analysis (lead); investigation (lead); validation (lead); visualization (lead); writing – original draft (lead). **Yuming Jia:** Data curation (equal); formal analysis (equal); investigation (equal); validation (equal); visualization (equal); writing – original draft (supporting). **Xiaowu Xu:** Investigation (equal); methodology (equal); software (equal); supervision (equal). **Yuheng Hu:** Investigation (equal); methodology (equal); software (equal); supervision (equal). **Guixiong Fan:** Investigation (equal); methodology (equal); supervision (equal). **Desheng Jing:** Investigation (equal); methodology (equal); supervision (equal). **Zhilei Zhang:** Investigation (equal); methodology (equal); supervision (equal). **Chao Wang:** Investigation (equal); methodology (equal); supervision (equal). **Changfeng Song:** Investigation (equal); methodology (equal); supervision (equal). **Yi Qin:** Conceptualization (equal); project administration (equal); resources (equal); writing – original draft (equal); writing – review and editing (equal). **Li Peng:** Conceptualization (lead); funding acquisition (lead); project administration (lead); supervision (lead); writing – original draft (equal); writing – review and editing (equal).

## FUNDING INFORMATION

The work was supported by the National Natural Science Foundation of China (No. 82173281) and Hebei Provincial Government Funded Special Funds for Clinical Medical Talents in 2021.

## CONFLICT OF INTEREST STATEMENT

The authors declare no conflict of interest.

## ETHICS STATEMENT

The study was approved by the Institutional Research Ethics Committee of Fudan University Shanghai Cancer Center. Informed consent was obtained from all subjects involved in the study.

## Supporting information


Table S1.

Table S2.

Table S3.

Table S4.

Table S5.

Table S6.

Table S7.

## Data Availability

The data that support the findings of this study are available from the corresponding author upon reasonable request.
