# Supplementary material for: Nuclear receptor coactivator 6 (NCoA6) promotes cell proliferation, migration, and invasion in pancreatic cancer
Source: Cancer Med. 2023 Aug 8;12(17):18425–39. doi: 10.1002/cam4.6427 (PMC10524018; doi:10.1002/cam4.6427)
Supplement: Supplementary file 1 — Table S1. [file CAM4-12-18425-s007.doc]

Supplementary Table 1. The DEGs that were significantly upregulated in the RNA-sequencing dataset of NCoA6-NC/sh PANC-1 cells.

| **Ensemble_ID** | **Symbol** | **log_2_FC** | ***P*value** | ***P*adj** |
| --- | --- | --- | --- | --- |
| ENSG00000197110 | IFNL3 | 9.270205869 | 7.15465E-15 | 5.14013E-13 |
| ENSG00000169245 | CXCL10 | 8.967602259 | 5.35775E-14 | 3.52779E-12 |
| ENSG00000137965 | IFI44 | 7.593874509 | 1.49051E-52 | 8.28575E-50 |
| ENSG00000268916 | CSAG3 | 7.549234823 | 3.42044E-10 | 1.40761E-08 |
| ENSG00000135114 | OASL | 7.500223026 | 7.3548E-219 | 6.235E-215 |
| ENSG00000182393 | IFNL1 | 7.456854614 | 1.87546E-37 | 5.67828E-35 |
| ENSG00000268902 | CSAG2 | 7.199382519 | 2.4222E-09 | 8.73797E-08 |
| ENSG00000134321 | RSAD2 | 7.073186977 | 5.84857E-21 | 7.00795E-19 |
| ENSG00000174808 | BTC | 6.957037482 | 6.17526E-09 | 2.07947E-07 |
| ENSG00000183709 | IFNL2 | 6.923333099 | 2.38504E-25 | 4.04384E-23 |
| ENSG00000137766 | UNC13C | 6.58723185 | 1.12628E-07 | 3.10002E-06 |
| ENSG00000169248 | CXCL11 | 6.552228982 | 1.85939E-10 | 8.0117E-09 |
| ENSG00000198535 | C2CD4A | 6.333538388 | 2.72769E-07 | 6.93892E-06 |
| ENSG00000163421 | PROK2 | 6.244279505 | 8.2634E-07 | 1.90232E-05 |
| ENSG00000134326 | CMPK2 | 6.089714942 | 7.1885E-41 | 2.64959E-38 |
| ENSG00000138135 | CH25H | 5.944764473 | 1.23494E-06 | 2.71575E-05 |
| ENSG00000077264 | PAK3 | 5.891460316 | 1.25537E-06 | 2.75354E-05 |
| ENSG00000095739 | BAMBI | 5.852223205 | 1.39754E-06 | 3.03398E-05 |
| ENSG00000272395 | IFNL4 | 5.780641596 | 3.93643E-06 | 7.75345E-05 |
| ENSG00000138646 | HERC5 | 5.733261076 | 7.3175E-283 | 2.4814E-278 |
| ENSG00000198797 | BRINP2 | 5.705999964 | 1.2776E-05 | 0.000221038 |
| ENSG00000273669 | AC015819.1 | 5.590158924 | 8.28907E-06 | 0.000150714 |
| ENSG00000121207 | LRAT | 5.572149549 | 2.67486E-17 | 2.33775E-15 |
| ENSG00000196611 | MMP1 | 5.567403956 | 8.28028E-43 | 3.26493E-40 |
| ENSG00000166396 | SERPINB7 | 5.510683086 | 2.60812E-07 | 6.66476E-06 |
| ENSG00000119917 | IFIT3 | 5.384799421 | 1.7182E-175 | 8.3235E-172 |
| ENSG00000249755 | AC083829.1 | 5.309291323 | 2.6112E-05 | 0.000411458 |
| ENSG00000164794 | KCNV1 | 5.253043246 | 1.75949E-05 | 0.00029062 |
| ENSG00000258733 | LINC02328 | 5.202646692 | 3.08283E-05 | 0.000477392 |
| ENSG00000145428 | RNF175 | 5.144637204 | 0.00020955 | 0.002594316 |
| ENSG00000172403 | SYNPO2 | 5.094377591 | 3.88843E-07 | 9.64571E-06 |
| ENSG00000116711 | PLA2G4A | 5.072355959 | 4.27914E-05 | 0.000632544 |
| ENSG00000119922 | IFIT2 | 5.047797734 | 1.103E-141 | 4.1558E-138 |
| ENSG00000133106 | EPSTI1 | 4.899283464 | 3.31266E-06 | 6.64689E-05 |
| ENSG00000112562 | SMOC2 | 4.865085545 | 0.000172282 | 0.002186411 |
| ENSG00000079841 | RIMS1 | 4.864087882 | 0.000184133 | 0.002318581 |
| ENSG00000154451 | GBP5 | 4.819819818 | 7.18649E-14 | 4.65954E-12 |
| ENSG00000101938 | CHRDL1 | 4.753569906 | 0.000153812 | 0.001977172 |
| ENSG00000164651 | SP8 | 4.740950137 | 0.000652417 | 0.006911426 |
| ENSG00000259256 | LINC01895 | 4.71748868 | 0.000541421 | 0.00589226 |
| ENSG00000164342 | TLR3 | 4.712511482 | 3.8083E-05 | 0.000572654 |
| ENSG00000168824 | NSG1 | 4.700544371 | 0.00031211 | 0.003668225 |
| ENSG00000073756 | PTGS2 | 4.663259555 | 7.90137E-07 | 1.83518E-05 |
| ENSG00000248740 | LINC02428 | 4.638018945 | 0.001092683 | 0.01069347 |
| ENSG00000251598 | AC093916.1 | 4.620487975 | 0.00048293 | 0.005348191 |
| ENSG00000012124 | CD22 | 4.576286383 | 2.19522E-05 | 0.000353015 |
| ENSG00000170006 | TMEM154 | 4.555879309 | 0.000327478 | 0.00382354 |
| ENSG00000135750 | KCNK1 | 4.516655081 | 0.000350104 | 0.004050508 |
| ENSG00000111335 | OAS2 | 4.508168082 | 3.15315E-63 | 2.48659E-60 |
| ENSG00000116701 | NCF2 | 4.491259895 | 0.000210542 | 0.002603391 |
| ENSG00000128422 | KRT17 | 4.420096594 | 0.000610115 | 0.006510066 |
| ENSG00000271503 | CCL5 | 4.327685234 | 1.8011E-239 | 3.0537E-235 |
| ENSG00000079215 | SLC1A3 | 4.312263221 | 0.000676713 | 0.007133147 |
| ENSG00000268089 | GABRQ | 4.311492123 | 6.17773E-05 | 0.000876514 |
| ENSG00000198963 | RORB | 4.310625557 | 7.16556E-05 | 0.000999523 |
| ENSG00000206432 | TMEM200C | 4.287597167 | 2.7029E-08 | 8.25723E-07 |
| ENSG00000168685 | IL7R | 4.25610129 | 0.000678496 | 0.007147498 |
| ENSG00000169116 | PARM1 | 4.253518956 | 1.77893E-99 | 4.02157E-96 |
| ENSG00000177822 | TENM3-AS1 | 4.224392604 | 0.00166932 | 0.015361371 |
| ENSG00000196104 | SPOCK3 | 4.212385017 | 1.32219E-26 | 2.35977E-24 |
| ENSG00000260418 | AL023284.4 | 4.211651374 | 0.002525913 | 0.021985041 |
| ENSG00000198626 | RYR2 | 4.178753087 | 0.000911044 | 0.009161776 |
| ENSG00000124107 | SLPI | 4.177346476 | 0.001377297 | 0.012976974 |
| ENSG00000272189 | AL024508.2 | 4.171983591 | 0.002871565 | 0.024383128 |
| ENSG00000086548 | CEACAM6 | 4.171187702 | 0.001356252 | 0.012807158 |
| ENSG00000108342 | CSF3 | 4.145081417 | 2.36744E-06 | 4.90408E-05 |
| ENSG00000182747 | SLC35D3 | 4.143590391 | 0.002742015 | 0.023509917 |
| ENSG00000165125 | TRPV6 | 4.106355292 | 3.049E-07 | 7.6757E-06 |
| ENSG00000171502 | COL24A1 | 4.076818075 | 0.001122446 | 0.01092484 |
| ENSG00000138795 | LEF1 | 4.069131912 | 4.541E-13 | 2.70437E-11 |
| ENSG00000182585 | EPGN | 4.069037042 | 0.002221316 | 0.01967733 |
| ENSG00000253130 | AC114550.1 | 4.051376529 | 0.006661797 | 0.049013137 |
| ENSG00000256916 | AP000851.1 | 4.031186542 | 0.004708045 | 0.036845096 |
| ENSG00000019549 | SNAI2 | 4.026765053 | 0.001801884 | 0.016407597 |
| ENSG00000125848 | FLRT3 | 3.997113232 | 2.59588E-22 | 3.4931E-20 |
| ENSG00000170989 | S1PR1 | 3.996787185 | 0.006797756 | 0.049829639 |
| ENSG00000105894 | PTN | 3.992838318 | 0.005435837 | 0.041515596 |
| ENSG00000046653 | GPM6B | 3.939513265 | 8.61391E-14 | 5.55319E-12 |
| ENSG00000288103 | AL359538.4 | 3.933558271 | 0.00644386 | 0.047647466 |
| ENSG00000010438 | PRSS3 | 3.906564903 | 4.71287E-21 | 5.66714E-19 |
| ENSG00000185745 | IFIT1 | 3.905006038 | 2.81E-177 | 1.5881E-173 |
| ENSG00000221867 | MAGEA3 | 3.903949904 | 0.002889002 | 0.024507153 |
| ENSG00000111962 | UST | 3.899849806 | 0.003468743 | 0.02859141 |
| ENSG00000140279 | DUOX2 | 3.884525546 | 1.57127E-11 | 7.83554E-10 |
| ENSG00000163412 | EIF4E3 | 3.875590713 | 1.31694E-12 | 7.54347E-11 |
| ENSG00000085552 | IGSF9 | 3.836997856 | 0.003456493 | 0.028504278 |
| ENSG00000136244 | IL6 | 3.829618522 | 0.000359251 | 0.004135162 |
| ENSG00000172572 | PDE3A | 3.81562533 | 0.003169152 | 0.026521691 |
| ENSG00000232618 | AL355304.1 | 3.806881451 | 0.000427829 | 0.004818228 |
| ENSG00000227028 | SLC8A1-AS1 | 3.791323095 | 0.006473254 | 0.0478231 |
| ENSG00000133107 | TRPC4 | 3.78707661 | 0.003964738 | 0.032056331 |
| ENSG00000100433 | KCNK10 | 3.774884945 | 1.1073E-10 | 4.93411E-09 |
| ENSG00000280294 | AC011008.2 | 3.774143491 | 0.00527064 | 0.040429428 |
| ENSG00000211772 | TRBC2 | 3.77369211 | 6.55039E-10 | 2.56198E-08 |
| ENSG00000104972 | LILRB1 | 3.773395545 | 0.00574285 | 0.043352635 |
| ENSG00000224014 | AL390728.3 | 3.753923887 | 0.005276781 | 0.040455715 |
| ENSG00000155918 | RAET1L | 3.750122803 | 0.000570773 | 0.006158096 |
| ENSG00000115267 | IFIH1 | 3.736559301 | 1.7547E-228 | 1.9834E-224 |
| ENSG00000230613 | HM13-AS1 | 3.730539944 | 0.006207388 | 0.046251929 |
| ENSG00000072041 | SLC6A15 | 3.723221132 | 4.18613E-21 | 5.0697E-19 |
| ENSG00000138642 | HERC6 | 3.705776548 | 3.6184E-21 | 4.44565E-19 |
| ENSG00000169436 | COL22A1 | 3.692584282 | 1.11792E-31 | 2.5614E-29 |
| ENSG00000285888 | Z85996.2 | 3.688989362 | 0.006105092 | 0.045589882 |
| ENSG00000105825 | TFPI2 | 3.68773785 | 2.54744E-24 | 4.01785E-22 |
| ENSG00000162654 | GBP4 | 3.679451048 | 1.97481E-38 | 6.439E-36 |
| ENSG00000137959 | IFI44L | 3.671703404 | 0.003544419 | 0.029144337 |
| ENSG00000115008 | IL1A | 3.665151924 | 0.004267129 | 0.03397472 |
| ENSG00000198483 | ANKRD35 | 3.658745833 | 4.96985E-06 | 9.55354E-05 |
| ENSG00000079308 | TNS1 | 3.648970878 | 0.004999174 | 0.038712492 |
| ENSG00000101955 | SRPX | 3.626099816 | 2.51841E-20 | 2.83719E-18 |
| ENSG00000118971 | CCND2 | 3.601923497 | 0.000133796 | 0.001757175 |
| ENSG00000225269 | LINC00705 | 3.56868579 | 0.005470214 | 0.041746787 |
| ENSG00000115602 | IL1RL1 | 3.559500183 | 6.98152E-10 | 2.72119E-08 |
| ENSG00000102755 | FLT1 | 3.557005552 | 0.004741881 | 0.037050044 |
| ENSG00000113430 | IRX4 | 3.541926495 | 3.67804E-07 | 9.16403E-06 |
| ENSG00000183023 | SLC8A1 | 3.535564407 | 1.00249E-56 | 6.29526E-54 |
| ENSG00000113532 | ST8SIA4 | 3.512486456 | 4.13724E-12 | 2.20935E-10 |
| ENSG00000058085 | LAMC2 | 3.509325982 | 1.61501E-30 | 3.60296E-28 |
| ENSG00000225206 | MIR137HG | 3.509272751 | 0.000130793 | 0.001721072 |
| ENSG00000287064 | AL606500.1 | 3.504016297 | 0.006331983 | 0.047025305 |
| ENSG00000153253 | SCN3A | 3.499626026 | 1.47221E-07 | 3.94335E-06 |
| ENSG00000128713 | HOXD11 | 3.480996856 | 0.006795255 | 0.049829285 |
| ENSG00000235750 | KIAA0040 | 3.444858499 | 6.08284E-16 | 4.74182E-14 |
| ENSG00000106123 | EPHB6 | 3.434654785 | 1.12388E-12 | 6.4814E-11 |
| ENSG00000170837 | GPR27 | 3.416933322 | 0.000154554 | 0.001982947 |
| ENSG00000130487 | KLHDC7B | 3.414315471 | 1.82444E-08 | 5.69152E-07 |
| ENSG00000115604 | IL18R1 | 3.387612806 | 5.60514E-08 | 1.62315E-06 |
| ENSG00000168016 | TRANK1 | 3.363740576 | 1.22937E-88 | 1.8949E-85 |
| ENSG00000173376 | NDNF | 3.32447021 | 0.001350419 | 0.012762736 |
| ENSG00000038295 | TLL1 | 3.319543298 | 2.14386E-09 | 7.78355E-08 |
| ENSG00000137628 | DDX60 | 3.299833717 | 2.5458E-94 | 4.796E-91 |
| ENSG00000152766 | ANKRD22 | 3.255837217 | 0.002876345 | 0.024414729 |
| ENSG00000169554 | ZEB2 | 3.253528634 | 3.26926E-23 | 4.97132E-21 |
| ENSG00000073282 | TP63 | 3.248953071 | 9.22257E-05 | 0.001255973 |
| ENSG00000135363 | LMO2 | 3.204379225 | 4.18685E-06 | 8.19724E-05 |
| ENSG00000164761 | TNFRSF11B | 3.186238269 | 2.20655E-06 | 4.61024E-05 |
| ENSG00000215182 | MUC5AC | 3.182045766 | 3.99566E-09 | 1.3911E-07 |
| ENSG00000187608 | ISG15 | 3.174472845 | 1.6623E-154 | 7.0459E-151 |
| ENSG00000172183 | ISG20 | 3.156943538 | 5.29865E-45 | 2.19118E-42 |
| ENSG00000133321 | PLAAT4 | 3.135961434 | 1.00946E-22 | 1.46285E-20 |
| ENSG00000141682 | PMAIP1 | 3.1242636 | 9.63775E-70 | 9.07823E-67 |
| ENSG00000008311 | AASS | 3.114906908 | 0.001103409 | 0.010754983 |
| ENSG00000188505 | NCCRP1 | 3.09540259 | 1.22881E-57 | 7.86208E-55 |
| ENSG00000268510 | IFNL3P1 | 3.07957458 | 0.000597078 | 0.006398545 |
| ENSG00000116132 | PRRX1 | 3.078409209 | 4.33432E-11 | 2.04703E-09 |
| ENSG00000164400 | CSF2 | 3.057947041 | 0.001205325 | 0.01159819 |
| ENSG00000073737 | DHRS9 | 3.057303715 | 0.004042352 | 0.032590623 |
| ENSG00000163661 | PTX3 | 3.054941024 | 2.97153E-11 | 1.42323E-09 |
| ENSG00000224417 | AL606970.1 | 3.04894 | 0.000120606 | 0.001602561 |
| ENSG00000102962 | CCL22 | 3.030446883 | 0.000264655 | 0.00318921 |
| ENSG00000167912 | LOC100505501 | 3.014540735 | 0.003988641 | 0.032211197 |
| ENSG00000120217 | CD274 | 2.996432194 | 1.77849E-07 | 4.7006E-06 |
| ENSG00000117228 | GBP1 | 2.98032878 | 4.94172E-12 | 2.60612E-10 |
| ENSG00000168243 | GNG4 | 2.977328582 | 3.60817E-09 | 1.26922E-07 |
| ENSG00000203697 | CAPN8 | 2.974021909 | 4.83893E-05 | 0.000702733 |
| ENSG00000163644 | PPM1K | 2.969800881 | 2.82789E-78 | 3.30668E-75 |
| ENSG00000150471 | ADGRL3 | 2.957848538 | 0.001052982 | 0.010370787 |
| ENSG00000180318 | ALX1 | 2.95322543 | 1.6344E-06 | 3.49448E-05 |
| ENSG00000153234 | NR4A2 | 2.92040684 | 4.3331E-19 | 4.3601E-17 |
| ENSG00000124882 | EREG | 2.904795207 | 2.75988E-17 | 2.39968E-15 |
| ENSG00000143507 | DUSP10 | 2.885554748 | 2.38342E-06 | 4.93417E-05 |
| ENSG00000179314 | WSCD1 | 2.881343426 | 1.51861E-05 | 0.000255563 |
| ENSG00000182836 | PLCXD3 | 2.859577543 | 1.95934E-15 | 1.45705E-13 |
| ENSG00000136826 | KLF4 | 2.858171637 | 8.61928E-29 | 1.71929E-26 |
| ENSG00000184588 | PDE4B | 2.843009232 | 8.69693E-14 | 5.59607E-12 |
| ENSG00000006210 | CX3CL1 | 2.841711208 | 6.12549E-98 | 1.29822E-94 |
| ENSG00000134363 | FST | 2.815984941 | 0.000522346 | 0.005726722 |
| ENSG00000213949 | ITGA1 | 2.814501947 | 2.19279E-22 | 3.01043E-20 |
| ENSG00000100867 | DHRS2 | 2.809914505 | 4.26597E-08 | 1.26119E-06 |
| ENSG00000203727 | SAMD5 | 2.807382511 | 5.75963E-10 | 2.27104E-08 |
| ENSG00000261618 | LINC02605 | 2.786530869 | 4.99505E-06 | 9.5913E-05 |
| ENSG00000197467 | COL13A1 | 2.778443832 | 1.88167E-22 | 2.61505E-20 |
| ENSG00000171951 | SCG2 | 2.723572722 | 4.142E-05 | 0.000615222 |
| ENSG00000163131 | CTSS | 2.723125206 | 3.95403E-21 | 4.82306E-19 |
| ENSG00000197172 | MAGEA6 | 2.721953455 | 0.000469955 | 0.00522326 |
| ENSG00000107201 | DDX58 | 2.721595351 | 3.94487E-94 | 7.04055E-91 |
| ENSG00000159167 | STC1 | 2.720763581 | 2.21322E-15 | 1.63865E-13 |
| ENSG00000135378 | PRRG4 | 2.710792748 | 5.31398E-26 | 9.38526E-24 |
| ENSG00000067798 | NAV3 | 2.676038974 | 1.56605E-61 | 1.18011E-58 |
| ENSG00000157064 | NMNAT2 | 2.672945087 | 1.40888E-06 | 3.05468E-05 |
| ENSG00000158352 | SHROOM4 | 2.672543846 | 4.81324E-07 | 1.16834E-05 |
| ENSG00000137752 | CASP1 | 2.664256352 | 2.24795E-10 | 9.56436E-09 |
| ENSG00000152049 | KCNE4 | 2.661583358 | 0.001442463 | 0.013497216 |
| ENSG00000011422 | PLAUR | 2.637376638 | 7.26247E-38 | 2.30159E-35 |
| ENSG00000204642 | HLA-F | 2.631984052 | 2.11712E-30 | 4.66179E-28 |
| ENSG00000167779 | IGFBP6 | 2.627316192 | 5.67629E-23 | 8.47943E-21 |
| ENSG00000105963 | ADAP1 | 2.606467685 | 1.83075E-15 | 1.36441E-13 |
| ENSG00000121742 | GJB6 | 2.600235862 | 9.41565E-17 | 7.86416E-15 |
| ENSG00000178764 | ZHX2 | 2.597061618 | 0.000246139 | 0.002995419 |
| ENSG00000227634 | LINC01714 | 2.586730627 | 0.00535534 | 0.040983883 |
| ENSG00000161405 | IKZF3 | 2.56939493 | 9.91816E-08 | 2.76583E-06 |
| ENSG00000205038 | PKHD1L1 | 2.568838213 | 1.54442E-10 | 6.72288E-09 |
| ENSG00000182985 | CADM1 | 2.562290006 | 8.7081E-50 | 4.34253E-47 |
| ENSG00000123610 | TNFAIP6 | 2.553004893 | 6.36537E-07 | 1.50309E-05 |
| ENSG00000144583 | MARCHF4 | 2.551526895 | 1.5886E-33 | 3.93207E-31 |
| ENSG00000023171 | GRAMD1B | 2.539275627 | 0.00188977 | 0.017102245 |
| ENSG00000085741 | WNT11 | 2.530639544 | 1.43983E-11 | 7.19065E-10 |
| ENSG00000188910 | GJB3 | 2.52498794 | 0.004610042 | 0.036228627 |
| ENSG00000049192 | ADAMTS6 | 2.521894931 | 0.004416414 | 0.03498262 |
| ENSG00000138821 | SLC39A8 | 2.50890635 | 1.54598E-13 | 9.76241E-12 |
| ENSG00000234745 | HLA-B | 2.502718576 | 3.0196E-204 | 2.0479E-200 |
| ENSG00000287023 | AC018978.1 | 2.499712467 | 0.000747482 | 0.007730132 |
| ENSG00000186088 | GSAP | 2.4960622 | 1.46515E-10 | 6.39422E-09 |
| ENSG00000173918 | C1QTNF1 | 2.495067123 | 1.70856E-11 | 8.4952E-10 |
| ENSG00000205220 | PSMB10 | 2.484959683 | 7.94902E-11 | 3.58923E-09 |
| ENSG00000198053 | SIRPA | 2.483422197 | 4.74535E-05 | 0.000690392 |
| ENSG00000143858 | SYT2 | 2.480480377 | 0.003050953 | 0.025663002 |
| ENSG00000111700 | SLCO1B3 | 2.476943425 | 3.61047E-14 | 2.42438E-12 |
| ENSG00000056558 | TRAF1 | 2.462495127 | 2.90062E-45 | 1.21432E-42 |
| ENSG00000276462 | LOC440896 | 2.457418889 | 0.006683874 | 0.049143573 |
| ENSG00000109321 | AREG | 2.450032696 | 1.20833E-07 | 3.30172E-06 |
| ENSG00000197943 | PLCG2 | 2.449049709 | 6.81974E-47 | 3.1251E-44 |
| ENSG00000148680 | HTR7 | 2.444891668 | 1.34678E-43 | 5.43684E-41 |
| ENSG00000271856 | LINC01215 | 2.437800962 | 0.004704598 | 0.036835123 |
| ENSG00000076356 | PLXNA2 | 2.4372023 | 0.000898607 | 0.009068894 |
| ENSG00000002587 | HS3ST1 | 2.434854258 | 4.01034E-12 | 2.14835E-10 |
| **ENSG00000165556** | **CDX2** | **2.429306148** | **5.12896E-13** | **6.68935E-11** |
| ENSG00000133019 | CHRM3 | 2.422250997 | 3.36972E-13 | 2.04414E-11 |
| ENSG00000169432 | SCN9A | 2.421703357 | 0.000913575 | 0.009181782 |
| ENSG00000169876 | MUC17 | 2.418692693 | 0.003825767 | 0.031103272 |
| ENSG00000177409 | SAMD9L | 2.409110341 | 7.0716E-11 | 3.21877E-09 |
| ENSG00000101076 | HNF4A | 2.393809372 | 0.002805014 | 0.023959203 |
| ENSG00000197249 | SERPINA1 | 2.386900491 | 2.09087E-07 | 5.44557E-06 |
| ENSG00000065618 | COL17A1 | 2.37918968 | 8.95384E-06 | 0.000161761 |
| ENSG00000100473 | COCH | 2.378355971 | 4.45637E-07 | 1.08951E-05 |
| ENSG00000237499 | WAKMAR2 | 2.374223026 | 0.005590036 | 0.042444721 |
| ENSG00000125845 | BMP2 | 2.361579128 | 0.000184796 | 0.002326073 |
| ENSG00000155629 | PIK3AP1 | 2.359428584 | 2.87826E-06 | 5.86198E-05 |
| ENSG00000102359 | SRPX2 | 2.35778465 | 4.51772E-06 | 8.77411E-05 |
| ENSG00000102362 | SYTL4 | 2.355834296 | 4.0102E-05 | 0.00059932 |
| ENSG00000255133 | LINC02721 | 2.355671168 | 0.000498074 | 0.005497945 |
| ENSG00000280303 | ERICD | 2.354772125 | 5.19485E-11 | 2.42975E-09 |
| ENSG00000143344 | RGL1 | 2.352353775 | 1.62144E-29 | 3.35262E-27 |
| ENSG00000196878 | LAMB3 | 2.349788856 | 2.22074E-07 | 5.72664E-06 |
| ENSG00000235881 | AC114776.1 | 2.342664007 | 1.72302E-11 | 8.55456E-10 |
| ENSG00000101298 | SNPH | 2.336185175 | 4.98244E-38 | 1.59391E-35 |
| ENSG00000038427 | VCAN | 2.335889254 | 0.000886746 | 0.008967955 |
| ENSG00000132274 | TRIM22 | 2.333400822 | 1.09512E-06 | 2.44474E-05 |
| ENSG00000185352 | HS6ST3 | 2.323860659 | 6.51813E-05 | 0.000919425 |
| ENSG00000249899 | AC025180.1 | 2.32308722 | 0.005414187 | 0.041378203 |
| ENSG00000110446 | SLC15A3 | 2.305687777 | 1.03575E-15 | 7.92825E-14 |
| ENSG00000221818 | EBF2 | 2.300587403 | 0.001755032 | 0.016032634 |
| ENSG00000114948 | ADAM23 | 2.29098548 | 0.000165142 | 0.002103673 |
| ENSG00000105855 | ITGB8 | 2.290085226 | 5.63967E-24 | 8.81296E-22 |
| ENSG00000243649 | CFB | 2.282495518 | 6.31738E-12 | 3.28059E-10 |
| ENSG00000174059 | CD34 | 2.279307445 | 0.000258976 | 0.003131911 |
| ENSG00000116741 | RGS2 | 2.25855928 | 2.18124E-10 | 9.30386E-09 |
| ENSG00000028277 | POU2F2 | 2.256023257 | 0.00100827 | 0.010000125 |
| ENSG00000077522 | ACTN2 | 2.249126429 | 3.22746E-06 | 6.49901E-05 |
| ENSG00000112299 | VNN1 | 2.243013438 | 0.000296753 | 0.003511133 |
| ENSG00000185532 | PRKG1 | 2.22336817 | 2.00406E-06 | 4.23677E-05 |
| ENSG00000113578 | FGF1 | 2.223269399 | 3.83918E-05 | 0.000576302 |
| ENSG00000164736 | SOX17 | 2.220058636 | 0.000529092 | 0.00578385 |
| ENSG00000138675 | FGF5 | 2.20798635 | 5.73229E-23 | 8.52552E-21 |
| ENSG00000082074 | FYB1 | 2.193507776 | 0.001554091 | 0.014420897 |
| ENSG00000164099 | PRSS12 | 2.190913346 | 0.001934395 | 0.017464148 |
| ENSG00000100342 | APOL1 | 2.190199256 | 8.09036E-82 | 9.79801E-79 |
| ENSG00000037280 | FLT4 | 2.164034401 | 0.000481047 | 0.005330817 |
| ENSG00000008056 | SYN1 | 2.161411954 | 0.004154951 | 0.033284759 |
| ENSG00000128284 | APOL3 | 2.149268967 | 6.24887E-20 | 6.76994E-18 |
| ENSG00000279443 | AL513497.1 | 2.141284189 | 0.003978 | 0.032148231 |
| ENSG00000169760 | NLGN1 | 2.133970043 | 3.31376E-22 | 4.40665E-20 |
| ENSG00000169418 | NPR1 | 2.133684784 | 0.000187656 | 0.002359442 |
| ENSG00000136514 | RTP4 | 2.118746657 | 6.11395E-09 | 2.06293E-07 |
| ENSG00000153294 | ADGRF4 | 2.105968925 | 0.000151397 | 0.001949075 |
| ENSG00000144481 | TRPM8 | 2.088884929 | 9.84485E-07 | 2.21967E-05 |
| ENSG00000067715 | SYT1 | 2.08009346 | 7.12191E-13 | 4.17105E-11 |
| ENSG00000166147 | FBN1 | 2.07437915 | 2.42194E-14 | 1.64916E-12 |
| ENSG00000205978 | NYNRIN | 2.070851751 | 0.002449475 | 0.021418693 |
| ENSG00000112379 | ARFGEF3 | 2.068247628 | 2.73582E-21 | 3.37352E-19 |
| ENSG00000049249 | TNFRSF9 | 2.068191017 | 8.03892E-07 | 1.86064E-05 |
| ENSG00000171246 | NPTX1 | 2.052755422 | 4.47283E-12 | 2.3699E-10 |
| ENSG00000127528 | KLF2 | 2.044883841 | 2.40221E-14 | 1.63901E-12 |
| ENSG00000003147 | ICA1 | 2.044201947 | 0.001096949 | 0.010716662 |
| ENSG00000277147 | LINC00869 | 2.038084437 | 0.00135512 | 0.012800037 |
| ENSG00000135525 | MAP7 | 2.037558638 | 3.68596E-09 | 1.29123E-07 |
| ENSG00000117226 | GBP3 | 2.036935114 | 1.96928E-27 | 3.68941E-25 |
| ENSG00000115738 | ID2 | 2.034926387 | 5.06862E-14 | 3.34391E-12 |
| ENSG00000105246 | EBI3 | 2.027417122 | 1.19377E-19 | 1.27297E-17 |
| ENSG00000225339 | AL354740.1 | 2.022040707 | 2.14759E-06 | 4.49814E-05 |
| ENSG00000059728 | MXD1 | 2.018360115 | 1.96401E-52 | 1.07419E-49 |
| ENSG00000267201 | LINC01775 | 2.016369004 | 0.003793364 | 0.03087685 |
| ENSG00000185070 | FLRT2 | 2.015684267 | 2.29151E-33 | 5.6308E-31 |
| ENSG00000135549 | PKIB | 2.008813929 | 0.001807727 | 0.016451963 |
| ENSG00000205755 | CRLF2 | 2.008430398 | 3.70228E-07 | 9.21764E-06 |
| ENSG00000164626 | KCNK5 | 1.99140561 | 7.78505E-07 | 1.81064E-05 |
| ENSG00000187595 | ZNF385C | 1.99086044 | 1.42823E-06 | 3.09069E-05 |
| ENSG00000003989 | SLC7A2 | 1.987297614 | 2.18326E-07 | 5.63857E-06 |
| ENSG00000006042 | TMEM98 | 1.985806524 | 0.000160343 | 0.00205179 |
| ENSG00000102760 | RGCC | 1.983367129 | 3.05775E-09 | 1.08347E-07 |
| ENSG00000285906 | AC083855.2 | 1.982329132 | 7.54368E-21 | 8.89457E-19 |
| ENSG00000231298 | MANCR | 1.975084411 | 9.59073E-05 | 0.001304016 |
| ENSG00000176771 | NCKAP5 | 1.974523564 | 0.001280985 | 0.012225786 |
| ENSG00000267505 | AC005180.2 | 1.970034752 | 0.001010644 | 0.010017808 |
| ENSG00000162511 | LAPTM5 | 1.967245473 | 0.004925381 | 0.0382196 |
| ENSG00000279821 | AC145098.2 | 1.963101095 | 5.56305E-05 | 0.000797645 |
| ENSG00000108771 | DHX58 | 1.957081397 | 4.5441E-18 | 4.22166E-16 |
| ENSG00000106541 | AGR2 | 1.951851594 | 2.69923E-05 | 0.000422776 |
| ENSG00000137462 | TLR2 | 1.951042668 | 0.001788838 | 0.0163107 |
| ENSG00000138449 | SLC40A1 | 1.950861121 | 0.004552735 | 0.035878046 |
| ENSG00000250697 | AC010343.3 | 1.944554884 | 0.004467743 | 0.03532319 |
| ENSG00000237943 | PRKCQ-AS1 | 1.937912143 | 0.000134244 | 0.001762336 |
| ENSG00000162510 | MATN1 | 1.93323279 | 0.00014215 | 0.001849832 |
| ENSG00000119508 | NR4A3 | 1.91912187 | 0.000285872 | 0.003409753 |
| ENSG00000135838 | NPL | 1.918208296 | 0.000375939 | 0.00429952 |
| ENSG00000144802 | NFKBIZ | 1.915110795 | 2.26643E-29 | 4.60207E-27 |
| ENSG00000079385 | CEACAM1 | 1.913415404 | 4.49664E-11 | 2.11486E-09 |
| ENSG00000231453 | LINC01305 | 1.908951626 | 0.003651043 | 0.029905042 |
| ENSG00000119699 | TGFB3 | 1.90719386 | 4.38625E-16 | 3.443E-14 |
| ENSG00000147394 | ZNF185 | 1.900607383 | 2.17195E-29 | 4.4368E-27 |
| ENSG00000196754 | S100A2 | 1.898772572 | 3.01694E-11 | 1.44294E-09 |
| ENSG00000261730 | AL034346.1 | 1.898353052 | 0.00376531 | 0.03068327 |
| ENSG00000174130 | TLR6 | 1.891869987 | 1.21793E-20 | 1.40956E-18 |
| ENSG00000164512 | ANKRD55 | 1.890914044 | 0.006423928 | 0.047541555 |
| ENSG00000129646 | QRICH2 | 1.881415472 | 0.004597501 | 0.036146828 |
| ENSG00000265787 | CYP4F35P | 1.874311931 | 0.00282669 | 0.024126119 |
| ENSG00000166401 | SERPINB8 | 1.871803813 | 3.37689E-12 | 1.82341E-10 |
| ENSG00000115009 | CCL20 | 1.868413214 | 3.40648E-16 | 2.68637E-14 |
| ENSG00000100055 | CYTH4 | 1.866186989 | 3.61019E-10 | 1.47496E-08 |
| ENSG00000287376 | LOC105370295 | 1.865810789 | 0.00468601 | 0.036715016 |
| ENSG00000173193 | PARP14 | 1.858592664 | 6.53611E-61 | 4.81825E-58 |
| ENSG00000168421 | RHOH | 1.856769298 | 0.000179514 | 0.002267155 |
| ENSG00000165935 | SMCO2 | 1.853254133 | 0.003209515 | 0.026793364 |
| ENSG00000103647 | CORO2B | 1.851634613 | 2.12791E-11 | 1.04461E-09 |
| ENSG00000185272 | RBM11 | 1.851155605 | 0.002966683 | 0.025037386 |
| ENSG00000232810 | TNF | 1.8429219 | 4.57734E-07 | 1.11667E-05 |
| ENSG00000055732 | MCOLN3 | 1.838838699 | 2.72653E-07 | 6.93892E-06 |
| ENSG00000164220 | F2RL2 | 1.8333704 | 0.000330204 | 0.003853142 |
| ENSG00000138685 | FGF2 | 1.831625076 | 8.53751E-31 | 1.93005E-28 |
| ENSG00000105499 | PLA2G4C | 1.831085541 | 3.974E-07 | 9.82921E-06 |
| ENSG00000163121 | NEURL3 | 1.830945349 | 1.51703E-05 | 0.000255424 |
| ENSG00000022267 | FHL1 | 1.825952019 | 9.7422E-25 | 1.59593E-22 |
| ENSG00000117020 | AKT3 | 1.824726954 | 9.76298E-40 | 3.41302E-37 |
| ENSG00000268001 | CARD8-AS1 | 1.82382561 | 0.00047126 | 0.005234334 |
| ENSG00000118503 | TNFAIP3 | 1.820355179 | 7.08608E-29 | 1.42183E-26 |
| ENSG00000170545 | SMAGP | 1.816975822 | 2.74157E-20 | 3.06821E-18 |
| ENSG00000146592 | CREB5 | 1.808982158 | 7.76705E-30 | 1.6359E-27 |
| ENSG00000135842 | NIBAN1 | 1.806761294 | 6.64776E-09 | 2.22973E-07 |
| ENSG00000162687 | KCNT2 | 1.805095092 | 0.005887677 | 0.044258727 |
| ENSG00000113070 | HBEGF | 1.803029354 | 6.44289E-34 | 1.65514E-31 |
| ENSG00000188897 | LOC400499 | 1.800524179 | 0.000510818 | 0.005618506 |
| ENSG00000127124 | HIVEP3 | 1.797733791 | 1.44348E-45 | 6.19601E-43 |
| ENSG00000224687 | RASAL2-AS1 | 1.796523226 | 1.6612E-06 | 3.54954E-05 |
| ENSG00000188641 | DPYD | 1.794486697 | 9.71808E-13 | 5.6622E-11 |
| ENSG00000064042 | LIMCH1 | 1.787714393 | 0.000899284 | 0.009069574 |
| ENSG00000188596 | CFAP54 | 1.785407126 | 4.50643E-05 | 0.000662102 |
| ENSG00000229677 | AC018644.1 | 1.783789813 | 3.33362E-06 | 6.67708E-05 |
| ENSG00000161544 | CYGB | 1.779386488 | 8.79352E-18 | 8.08098E-16 |
| ENSG00000196092 | PAX5 | 1.77615055 | 3.49365E-06 | 6.96881E-05 |
| ENSG00000111249 | CUX2 | 1.762871685 | 1.53515E-14 | 1.06674E-12 |
| ENSG00000172594 | SMPDL3A | 1.761394426 | 4.27938E-10 | 1.72549E-08 |
| ENSG00000151651 | ADAM8 | 1.760917163 | 2.84586E-06 | 5.80296E-05 |
| ENSG00000152689 | RASGRP3 | 1.753732564 | 1.01066E-14 | 7.13987E-13 |
| ENSG00000231728 | TMSB15B-AS1 | 1.753694269 | 0.002552822 | 0.022187618 |
| ENSG00000218336 | TENM3 | 1.735752467 | 1.14083E-06 | 2.53509E-05 |
| ENSG00000265298 | LOC101929240 | 1.730978759 | 0.003981963 | 0.032172291 |
| ENSG00000164488 | DACT2 | 1.728769287 | 6.06244E-50 | 3.06832E-47 |
| ENSG00000275202 | AL161421.1 | 1.716998351 | 0.0014814 | 0.013812006 |
| ENSG00000131378 | RFTN1 | 1.714974885 | 0.000610564 | 0.006512812 |
| ENSG00000106070 | GRB10 | 1.712557622 | 3.84561E-36 | 1.11457E-33 |
| ENSG00000229474 | PATL2 | 1.712041467 | 0.004847323 | 0.037682884 |
| ENSG00000125730 | C3 | 1.707780157 | 4.19665E-11 | 1.98755E-09 |
| ENSG00000111961 | SASH1 | 1.706945077 | 4.90377E-49 | 2.37553E-46 |
| ENSG00000127325 | BEST3 | 1.703724813 | 0.005616936 | 0.042620344 |
| ENSG00000163508 | EOMES | 1.701816936 | 8.34868E-10 | 3.21709E-08 |
| ENSG00000174125 | TLR1 | 1.699564235 | 1.47285E-05 | 0.000248794 |
| ENSG00000135318 | NT5E | 1.699474212 | 1.47289E-90 | 2.37837E-87 |
| ENSG00000158560 | DYNC1I1 | 1.698424675 | 0.00012859 | 0.001697133 |
| ENSG00000147571 | CRH | 1.698083436 | 0.000829662 | 0.008466402 |
| ENSG00000169896 | ITGAM | 1.696172617 | 1.74178E-06 | 3.70538E-05 |
| ENSG00000235978 | AC018816.1 | 1.693891435 | 2.90986E-06 | 5.91211E-05 |
| ENSG00000131459 | GFPT2 | 1.692947841 | 1.97016E-43 | 7.85979E-41 |
| ENSG00000151023 | ENKUR | 1.692937004 | 0.002854972 | 0.024297857 |
| ENSG00000139209 | SLC38A4 | 1.687983328 | 2.29992E-14 | 1.57556E-12 |
| ENSG00000230521 | AL645929.1 | 1.67436452 | 0.002200011 | 0.019508987 |
| ENSG00000164683 | HEY1 | 1.671536013 | 6.95784E-12 | 3.59118E-10 |
| ENSG00000159713 | TPPP3 | 1.663300805 | 0.000305661 | 0.003601445 |
| ENSG00000140379 | BCL2A1 | 1.662103509 | 2.19868E-28 | 4.28491E-26 |
| ENSG00000283294 | AP005212.4 | 1.658036766 | 0.000732932 | 0.007607505 |
| ENSG00000117318 | ID3 | 1.654881169 | 6.36699E-05 | 0.000900353 |
| ENSG00000100628 | ASB2 | 1.654828256 | 0.00010035 | 0.001354641 |
| ENSG00000254615 | AC027031.2 | 1.650039101 | 2.24512E-05 | 0.000360133 |
| ENSG00000073849 | ST6GAL1 | 1.648456757 | 4.38978E-06 | 8.55376E-05 |
| ENSG00000288011 | LOC283045 | 1.642479296 | 0.001312525 | 0.01247763 |
| ENSG00000174521 | TTC9B | 1.641700663 | 1.87193E-05 | 0.000307098 |
| ENSG00000285744 | AC083837.1 | 1.639355222 | 0.000819343 | 0.008373698 |
| ENSG00000186854 | TRABD2A | 1.63693628 | 3.02935E-06 | 6.12553E-05 |
| ENSG00000166750 | SLFN5 | 1.631372022 | 1.41422E-06 | 3.06234E-05 |
| ENSG00000272123 | AC008966.2 | 1.629826028 | 3.92368E-05 | 0.000587685 |
| ENSG00000152784 | PRDM8 | 1.627722999 | 9.50333E-36 | 2.731E-33 |
| ENSG00000145708 | CRHBP | 1.626985951 | 0.002666304 | 0.023004074 |
| ENSG00000157601 | MX1 | 1.624017744 | 2.99815E-17 | 2.58695E-15 |
| ENSG00000126709 | IFI6 | 1.618884198 | 1.9239E-18 | 1.84292E-16 |
| ENSG00000165474 | GJB2 | 1.616306066 | 6.07602E-21 | 7.25486E-19 |
| ENSG00000111181 | SLC6A12 | 1.615023825 | 0.000530154 | 0.005788994 |
| ENSG00000181381 | DDX60L | 1.614661508 | 1.20261E-25 | 2.08063E-23 |
| ENSG00000286048 | AC008966.3 | 1.600349235 | 0.000640116 | 0.006791721 |
| ENSG00000185338 | SOCS1 | 1.600277841 | 1.12284E-05 | 0.000198103 |
| ENSG00000170955 | CAVIN3 | 1.599047574 | 2.29886E-11 | 1.12003E-09 |
| ENSG00000100505 | TRIM9 | 1.595648331 | 0.000501934 | 0.005533347 |
| ENSG00000112406 | HECA | 1.59224553 | 7.35476E-22 | 9.62934E-20 |
| ENSG00000135333 | EPHA7 | 1.589985232 | 1.84263E-21 | 2.32281E-19 |
| ENSG00000226887 | ERVMER34-1 | 1.588965243 | 6.04619E-05 | 0.000858929 |
| ENSG00000271781 | AC026740.1 | 1.585905657 | 0.006074637 | 0.045382451 |
| ENSG00000164171 | ITGA2 | 1.58533877 | 1.11417E-83 | 1.45314E-80 |
| ENSG00000125347 | IRF1 | 1.581629592 | 1.13846E-39 | 3.9393E-37 |
| ENSG00000146386 | ABRACL | 1.579498363 | 3.44067E-25 | 5.74744E-23 |
| ENSG00000167244 | IGF2 | 1.574663997 | 0.001600618 | 0.014785335 |
| ENSG00000005981 | ASB4 | 1.571958654 | 3.36982E-10 | 1.38846E-08 |
| ENSG00000156804 | FBXO32 | 1.56906647 | 1.09728E-18 | 1.0723E-16 |
| ENSG00000234883 | MIR155HG | 1.56828941 | 5.04806E-05 | 0.000729045 |
| ENSG00000205177 | C11orf91 | 1.567641179 | 0.000502673 | 0.005536099 |
| ENSG00000206344 | HCG27 | 1.567529304 | 0.000190385 | 0.00239021 |
| ENSG00000101842 | VSIG1 | 1.56316627 | 5.99626E-05 | 0.000852193 |
| ENSG00000110852 | CLEC2B | 1.560528652 | 5.70415E-09 | 1.93815E-07 |
| ENSG00000163874 | ZC3H12A | 1.560300797 | 1.69327E-18 | 1.63586E-16 |
| ENSG00000151014 | NOCT | 1.555320728 | 2.90728E-25 | 4.88049E-23 |
| ENSG00000248323 | LUCAT1 | 1.554625913 | 7.3006E-25 | 1.20763E-22 |
| ENSG00000145536 | ADAMTS16 | 1.551388379 | 5.61575E-07 | 1.34106E-05 |
| ENSG00000010030 | ETV7 | 1.54941458 | 4.4308E-05 | 0.00065212 |
| ENSG00000150630 | VEGFC | 1.54451224 | 9.73832E-38 | 3.05765E-35 |
| ENSG00000026103 | FAS | 1.543667441 | 1.2761E-37 | 3.93388E-35 |
| ENSG00000215861 | AC245297.1 | 1.541472398 | 0.000608571 | 0.00649564 |
| ENSG00000179148 | ALOXE3 | 1.538152996 | 0.001324204 | 0.012567521 |
| ENSG00000154734 | ADAMTS1 | 1.537281706 | 0.000585767 | 0.006291843 |
| ENSG00000006468 | ETV1 | 1.536403655 | 2.10742E-06 | 4.43041E-05 |
| ENSG00000244159 | RPS27AP13 | 1.534342221 | 0.004663093 | 0.036560802 |
| ENSG00000105559 | PLEKHA4 | 1.529031731 | 1.64697E-15 | 1.24109E-13 |
| ENSG00000137673 | MMP7 | 1.528488088 | 8.35618E-06 | 0.000151691 |
| ENSG00000130222 | GADD45G | 1.527773672 | 0.004272949 | 0.034011338 |
| ENSG00000248719 | AC021127.1 | 1.52718466 | 0.000193875 | 0.002426842 |
| ENSG00000253522 | MIR3142HG | 1.522522921 | 8.04391E-07 | 1.86064E-05 |
| ENSG00000135678 | CPM | 1.52023376 | 0.000230836 | 0.002823825 |
| ENSG00000132357 | CARD6 | 1.520222107 | 5.35491E-07 | 1.28692E-05 |
| ENSG00000184226 | PCDH9 | 1.519743377 | 1.23559E-14 | 8.65676E-13 |
| ENSG00000088538 | DOCK3 | 1.517545271 | 1.45186E-05 | 0.000245549 |
| ENSG00000064300 | NGFR | 1.515699656 | 1.1419E-06 | 2.5358E-05 |
| ENSG00000280693 | SH3PXD2A-AS1 | 1.510886781 | 0.001423956 | 0.013342455 |
| ENSG00000147852 | VLDLR | 1.507846081 | 3.36704E-12 | 1.82099E-10 |
| ENSG00000138678 | GPAT3 | 1.504890036 | 5.63211E-33 | 1.3545E-30 |
| ENSG00000157657 | ZNF618 | 1.504233372 | 0.00498527 | 0.038622461 |
| ENSG00000167550 | RHEBL1 | 1.49381842 | 1.31911E-09 | 4.93719E-08 |
| ENSG00000130477 | UNC13A | 1.49079114 | 9.31674E-08 | 2.60669E-06 |
| ENSG00000100906 | NFKBIA | 1.48889154 | 1.47179E-22 | 2.07952E-20 |
| ENSG00000158050 | DUSP2 | 1.487459461 | 1.87736E-05 | 0.000307692 |
| ENSG00000158077 | NLRP14 | 1.486647114 | 0.005658485 | 0.042841355 |
| ENSG00000231131 | LOC105378305 | 1.479122743 | 0.000212356 | 0.002623312 |
| ENSG00000275793 | RIMBP3 | 1.474677709 | 0.001359469 | 0.012830387 |
| ENSG00000118473 | SGIP1 | 1.473207169 | 5.33215E-10 | 2.11556E-08 |
| ENSG00000103044 | HAS3 | 1.472116195 | 3.4295E-14 | 2.30743E-12 |
| ENSG00000188760 | TMEM198 | 1.471401544 | 0.00094423 | 0.009456889 |
| ENSG00000274721 | AC091825.1 | 1.470054795 | 5.47905E-06 | 0.000104673 |
| ENSG00000064886 | CHI3L2 | 1.468857038 | 2.28095E-07 | 5.86408E-06 |
| ENSG00000228835 | AC012123.1 | 1.463956556 | 0.00039747 | 0.004516825 |
| ENSG00000267121 | LOC339192 | 1.458526496 | 9.94381E-05 | 0.001345012 |
| ENSG00000168389 | MFSD2A | 1.456500068 | 1.41706E-07 | 3.81066E-06 |
| ENSG00000111801 | BTN3A3 | 1.454499368 | 3.78415E-10 | 1.53677E-08 |
| ENSG00000104267 | CA2 | 1.453182417 | 5.73254E-11 | 2.64477E-09 |
| ENSG00000287839 | AL353807.5 | 1.453105635 | 7.9796E-10 | 3.08891E-08 |
| ENSG00000196923 | PDLIM7 | 1.451146658 | 1.72288E-32 | 4.0855E-30 |
| ENSG00000105939 | ZC3HAV1 | 1.449806066 | 3.69617E-64 | 3.057E-61 |
| ENSG00000162545 | CAMK2N1 | 1.448713082 | 0.002776542 | 0.023755946 |
| ENSG00000198488 | B3GNT6 | 1.446913703 | 0.000249376 | 0.003027691 |
| ENSG00000161270 | NPHS1 | 1.443914557 | 1.61595E-05 | 0.000269802 |
| ENSG00000008517 | IL32 | 1.432699564 | 5.46682E-14 | 3.59263E-12 |
| ENSG00000081041 | CXCL2 | 1.432434671 | 2.13568E-05 | 0.000344862 |
| ENSG00000210176 | MT-TH | 1.426972546 | 1.97759E-08 | 6.10192E-07 |
| ENSG00000131015 | ULBP2 | 1.42512757 | 0.001156444 | 0.011207663 |
| ENSG00000104951 | IL4I1 | 1.421734255 | 1.01836E-06 | 2.28843E-05 |
| ENSG00000232327 | AC105917.1 | 1.418880229 | 0.000110762 | 0.001482801 |
| ENSG00000071054 | MAP4K4 | 1.407372581 | 5.21593E-64 | 4.21124E-61 |
| ENSG00000198848 | CES1 | 1.403853352 | 8.29361E-21 | 9.69781E-19 |
| ENSG00000121454 | LHX4 | 1.401605805 | 5.50908E-09 | 1.87752E-07 |
| ENSG00000230453 | ANKRD18B | 1.393484583 | 2.08483E-08 | 6.42698E-07 |
| ENSG00000277013 | AC008556.1 | 1.39060769 | 0.00237413 | 0.020835077 |
| ENSG00000101384 | JAG1 | 1.388390655 | 3.19569E-17 | 2.74681E-15 |
| ENSG00000225886 | AL445490.1 | 1.387500001 | 0.006279719 | 0.046719014 |
| ENSG00000116729 | WLS | 1.387137936 | 1.21035E-34 | 3.20648E-32 |
| ENSG00000138166 | DUSP5 | 1.382467878 | 6.24588E-13 | 3.68344E-11 |
| ENSG00000183840 | GPR39 | 1.376617485 | 6.76546E-10 | 2.64301E-08 |
| ENSG00000219891 | ZSCAN12P1 | 1.374099043 | 0.001744926 | 0.015953202 |
| ENSG00000104856 | RELB | 1.373321499 | 7.49897E-26 | 1.31077E-23 |
| ENSG00000023445 | BIRC3 | 1.372496526 | 4.84609E-31 | 1.10289E-28 |
| ENSG00000100558 | PLEK2 | 1.366389401 | 6.65126E-20 | 7.18294E-18 |
| ENSG00000144655 | CSRNP1 | 1.365640364 | 1.79582E-17 | 1.58584E-15 |
| ENSG00000060982 | BCAT1 | 1.365412833 | 0.002863913 | 0.024345772 |
| ENSG00000117983 | MUC5B | 1.362395509 | 0.001535482 | 0.014280913 |
| ENSG00000089723 | OTUB2 | 1.36168055 | 7.8239E-11 | 3.54217E-09 |
| ENSG00000174804 | FZD4 | 1.360975522 | 1.01991E-20 | 1.18849E-18 |
| ENSG00000123358 | NR4A1 | 1.354524875 | 9.82814E-07 | 2.21738E-05 |
| ENSG00000204525 | HLA-C | 1.348654714 | 4.82432E-63 | 3.71802E-60 |
| ENSG00000168386 | FILIP1L | 1.34770044 | 1.31368E-09 | 4.92229E-08 |
| ENSG00000197705 | KLHL14 | 1.346069785 | 7.94709E-41 | 2.8977E-38 |
| ENSG00000164181 | ELOVL7 | 1.345769421 | 5.32385E-11 | 2.48666E-09 |
| ENSG00000249992 | TMEM158 | 1.345425874 | 0.002452208 | 0.02143154 |
| ENSG00000182179 | UBA7 | 1.343776003 | 5.44657E-07 | 1.30525E-05 |
| ENSG00000149380 | P4HA3 | 1.342327898 | 7.40322E-08 | 2.09727E-06 |
| ENSG00000124145 | SDC4 | 1.341528955 | 8.36087E-07 | 1.92085E-05 |
| ENSG00000248019 | FAM13A-AS1 | 1.338366166 | 0.000268909 | 0.003232438 |
| ENSG00000058091 | CDK14 | 1.338308836 | 7.70643E-07 | 1.79358E-05 |
| ENSG00000128335 | APOL2 | 1.338035474 | 4.45329E-58 | 2.961E-55 |
| ENSG00000101457 | DNTTIP1 | 1.337686754 | 7.37765E-23 | 1.08772E-20 |
| ENSG00000170214 | ADRA1B | 1.337642923 | 0.002643386 | 0.022843329 |
| ENSG00000234155 | LINC02535 | 1.33568427 | 0.005772804 | 0.043501284 |
| ENSG00000177606 | JUN | 1.334344482 | 2.28809E-73 | 2.35119E-70 |
| ENSG00000160886 | LY6K | 1.326153714 | 0.001080548 | 0.010593061 |
| ENSG00000184979 | USP18 | 1.325792201 | 1.02302E-16 | 8.48185E-15 |
| ENSG00000057704 | TMCC3 | 1.325380509 | 0.003152331 | 0.02639396 |
| ENSG00000272711 | AC019069.1 | 1.324870454 | 4.74581E-05 | 0.000690392 |
| ENSG00000188158 | NHS | 1.324772608 | 2.66585E-09 | 9.5559E-08 |
| ENSG00000223947 | AC016738.1 | 1.323547637 | 0.000624566 | 0.006651708 |
| ENSG00000168079 | SCARA5 | 1.321662861 | 0.000126479 | 0.001672737 |
| ENSG00000221887 | HMSD | 1.32003174 | 0.000117261 | 0.001563019 |
| ENSG00000102554 | KLF5 | 1.319000591 | 1.34562E-15 | 1.0254E-13 |
| ENSG00000164379 | FOXQ1 | 1.318781751 | 2.71445E-17 | 2.36625E-15 |
| ENSG00000145703 | IQGAP2 | 1.316043323 | 0.000125994 | 0.001666978 |
| ENSG00000131781 | FMO5 | 1.313801488 | 0.001045633 | 0.010316384 |
| ENSG00000198513 | ATL1 | 1.30967309 | 0.000274804 | 0.003291635 |
| ENSG00000272841 | AL139393.3 | 1.308573648 | 2.13499E-07 | 5.5221E-06 |
| ENSG00000145476 | CYP4V2 | 1.307561322 | 0.002050817 | 0.018358817 |
| ENSG00000230795 | HLA-K | 1.305438327 | 4.50974E-06 | 8.76363E-05 |
| ENSG00000089127 | OAS1 | 1.303895409 | 5.26687E-07 | 1.26846E-05 |
| ENSG00000135604 | STX11 | 1.298540126 | 2.58399E-27 | 4.74425E-25 |
| ENSG00000139211 | AMIGO2 | 1.297700296 | 1.39162E-20 | 1.59966E-18 |
| ENSG00000136158 | SPRY2 | 1.295391211 | 5.50977E-50 | 2.83085E-47 |
| ENSG00000196503 | ARL9 | 1.295038456 | 0.004236373 | 0.03381719 |
| ENSG00000105852 | PON3 | 1.293052155 | 1.73012E-07 | 4.59065E-06 |
| ENSG00000145901 | TNIP1 | 1.29001086 | 1.11487E-33 | 2.80039E-31 |
| ENSG00000278993 | AC002350.2 | 1.288485554 | 0.004396317 | 0.034839712 |
| ENSG00000226067 | LINC00623 | 1.28620693 | 1.11539E-07 | 3.07538E-06 |
| ENSG00000285844 | FO393414.3 | 1.28595971 | 2.1285E-07 | 5.51393E-06 |
| ENSG00000028137 | TNFRSF1B | 1.285715533 | 0.000345524 | 0.004004345 |
| ENSG00000161249 | DMKN | 1.284761602 | 0.002004709 | 0.017998325 |
| ENSG00000196639 | HRH1 | 1.281573131 | 1.08257E-59 | 7.64793E-57 |
| ENSG00000129595 | EPB41L4A | 1.269535864 | 0.004825336 | 0.037520556 |
| ENSG00000151702 | FLI1 | 1.265749935 | 0.004783794 | 0.037265896 |
| ENSG00000104332 | SFRP1 | 1.263752691 | 5.66922E-53 | 3.25836E-50 |
| ENSG00000143013 | LMO4 | 1.259989949 | 1.80277E-42 | 6.94679E-40 |
| ENSG00000118402 | ELOVL4 | 1.257297036 | 8.45643E-06 | 0.000153264 |
| ENSG00000285867 | BX470102.2 | 1.256243176 | 0.004805435 | 0.037408699 |
| ENSG00000163710 | PCOLCE2 | 1.251147698 | 4.7148E-06 | 9.12551E-05 |
| ENSG00000099284 | MACROH2A2 | 1.250413992 | 0.005892208 | 0.044273163 |
| ENSG00000170396 | ZNF804A | 1.248891509 | 6.12998E-13 | 3.62139E-11 |
| ENSG00000163659 | TIPARP | 1.246478366 | 7.52692E-21 | 8.89457E-19 |
| ENSG00000166313 | APBB1 | 1.245988936 | 5.98854E-07 | 1.42207E-05 |
| ENSG00000287778 | AC006230.1 | 1.244128188 | 0.000213285 | 0.002632175 |
| ENSG00000235052 | AL021154.1 | 1.2435098 | 0.006014809 | 0.045004893 |
| ENSG00000196352 | CD55 | 1.239798175 | 5.77373E-12 | 3.03076E-10 |
| ENSG00000185022 | MAFF | 1.239094146 | 3.64818E-23 | 5.52275E-21 |
| ENSG00000163132 | MSX1 | 1.237958429 | 3.60269E-06 | 7.16103E-05 |
| ENSG00000172602 | RND1 | 1.234174472 | 0.001658778 | 0.015272652 |
| ENSG00000204301 | NOTCH4 | 1.230117046 | 4.47251E-08 | 1.31881E-06 |
| ENSG00000176658 | MYO1D | 1.224944863 | 1.35224E-07 | 3.65374E-06 |
| ENSG00000261253 | AC137932.2 | 1.221986075 | 0.000360345 | 0.004144947 |
| ENSG00000172738 | TMEM217 | 1.219271282 | 0.005472264 | 0.041746787 |
| ENSG00000196628 | TCF4 | 1.218159909 | 1.11662E-05 | 0.000197261 |
| ENSG00000157873 | TNFRSF14 | 1.217281894 | 9.78094E-13 | 5.68905E-11 |
| ENSG00000249242 | TMEM150C | 1.21574373 | 0.003950658 | 0.031980617 |
| ENSG00000127507 | ADGRE2 | 1.215173759 | 0.000447767 | 0.005007843 |
| ENSG00000181773 | GPR3 | 1.209215581 | 2.22742E-05 | 0.000357632 |
| ENSG00000174899 | SLC66A1L | 1.207474735 | 0.000145327 | 0.001885419 |
| ENSG00000260277 | AC009019.1 | 1.201529851 | 0.005165814 | 0.039784866 |
| ENSG00000105889 | LOC401312 | 1.20007491 | 1.69506E-08 | 5.3331E-07 |
| ENSG00000244588 | RAD21L1 | 1.1965126 | 4.61946E-05 | 0.00067578 |
| ENSG00000205730 | ITPRIPL2 | 1.196405772 | 1.54227E-40 | 5.50508E-38 |
| ENSG00000132530 | XAF1 | 1.196337435 | 0.000156732 | 0.002008608 |
| ENSG00000101670 | LIPG | 1.19501623 | 9.26355E-07 | 2.10541E-05 |
| ENSG00000133121 | STARD13 | 1.192568548 | 1.86135E-21 | 2.33771E-19 |
| ENSG00000128596 | CCDC136 | 1.192537544 | 0.00014216 | 0.001849832 |
| ENSG00000164509 | IL31RA | 1.19252847 | 2.91742E-09 | 1.037E-07 |
| ENSG00000145362 | ANK2 | 1.192407572 | 1.46694E-08 | 4.67519E-07 |
| ENSG00000177432 | NAP1L5 | 1.191025941 | 6.48498E-06 | 0.000121428 |
| ENSG00000109738 | GLRB | 1.189368274 | 0.00486875 | 0.037823442 |
| ENSG00000153993 | SEMA3D | 1.187266397 | 6.31051E-07 | 1.49226E-05 |
| ENSG00000198168 | SVIP | 1.18724262 | 1.9135E-13 | 1.19497E-11 |
| ENSG00000143147 | GPR161 | 1.184989293 | 0.002325988 | 0.020483222 |
| ENSG00000187867 | PALM3 | 1.184702965 | 0.000115241 | 0.001539116 |
| ENSG00000111331 | OAS3 | 1.183880497 | 9.06921E-47 | 4.04654E-44 |
| ENSG00000134755 | DSC2 | 1.183463739 | 5.19544E-08 | 1.51225E-06 |
| ENSG00000197852 | INKA2 | 1.17966321 | 6.23653E-05 | 0.000883747 |
| ENSG00000174607 | UGT8 | 1.178763591 | 0.001255421 | 0.012015059 |
| ENSG00000007908 | SELE | 1.178292014 | 0.00613968 | 0.045777605 |
| ENSG00000121858 | TNFSF10 | 1.17764384 | 3.83517E-06 | 7.58312E-05 |
| ENSG00000116514 | RNF19B | 1.17578242 | 3.50577E-46 | 1.52411E-43 |
| ENSG00000049089 | COL9A2 | 1.174346278 | 9.53041E-07 | 2.15738E-05 |
| ENSG00000162458 | FBLIM1 | 1.171514057 | 2.46652E-05 | 0.000391205 |
| ENSG00000210741 | MIR196A1 | 1.167084833 | 0.003950547 | 0.031980617 |
| ENSG00000121281 | ADCY7 | 1.165002988 | 1.42109E-09 | 5.2781E-08 |
| ENSG00000171444 | MCC | 1.160399634 | 7.56203E-05 | 0.001048359 |
| ENSG00000266094 | RASSF5 | 1.155258531 | 4.16551E-05 | 0.000618444 |
| ENSG00000178685 | PARP10 | 1.154080783 | 4.10332E-21 | 4.98723E-19 |
| ENSG00000246763 | RGMB-AS1 | 1.15265782 | 0.003161945 | 0.026467924 |
| ENSG00000227507 | LTB | 1.151441188 | 2.94529E-07 | 7.44095E-06 |
| ENSG00000163288 | GABRB1 | 1.150492181 | 1.52903E-07 | 4.08264E-06 |
| ENSG00000275216 | AL161431.1 | 1.150139925 | 3.1061E-10 | 1.28763E-08 |
| ENSG00000198814 | GK | 1.149552332 | 4.23637E-09 | 1.47037E-07 |
| ENSG00000196275 | GTF2IRD2 | 1.147952897 | 0.004149357 | 0.033263522 |
| ENSG00000071242 | RPS6KA2 | 1.145288325 | 6.39222E-05 | 0.000903168 |
| ENSG00000168062 | BATF2 | 1.145283635 | 0.000235664 | 0.002880805 |
| ENSG00000165891 | E2F7 | 1.144900726 | 7.08819E-16 | 5.51285E-14 |
| ENSG00000188921 | HACD4 | 1.142204684 | 0.004706737 | 0.036843362 |
| ENSG00000177842 | ZNF620 | 1.142117595 | 6.39009E-05 | 0.000903168 |
| ENSG00000214783 | POLR2J4 | 1.141454861 | 0.001224992 | 0.01175757 |
| ENSG00000260430 | AC099518.1 | 1.141119206 | 0.001034777 | 0.010221175 |
| ENSG00000125285 | SOX21 | 1.140659753 | 7.37289E-11 | 3.34244E-09 |
| ENSG00000144821 | MYH15 | 1.139893586 | 0.000227821 | 0.002790976 |
| ENSG00000135919 | SERPINE2 | 1.139200933 | 0.00012661 | 0.001673815 |
| ENSG00000128394 | APOBEC3F | 1.138267541 | 8.97932E-11 | 4.02763E-09 |
| ENSG00000183018 | SPNS2 | 1.136380019 | 0.000143129 | 0.001859587 |
| ENSG00000240288 | GHRLOS | 1.134036746 | 0.000551815 | 0.00598211 |
| ENSG00000109861 | CTSC | 1.133121915 | 2.37627E-22 | 3.22317E-20 |
| ENSG00000187678 | SPRY4 | 1.131687976 | 1.13215E-48 | 5.40723E-46 |
| ENSG00000132109 | TRIM21 | 1.131665496 | 2.03918E-27 | 3.79938E-25 |
| ENSG00000069667 | RORA | 1.124327759 | 3.11307E-05 | 0.000481809 |
| ENSG00000167105 | TMEM92 | 1.123967073 | 0.001983202 | 0.017828839 |
| ENSG00000091986 | CCDC80 | 1.121777182 | 0.004088126 | 0.032873693 |
| ENSG00000130589 | HELZ2 | 1.121254443 | 3.75747E-34 | 9.87721E-32 |
| ENSG00000148057 | IDNK | 1.119880263 | 5.9002E-05 | 0.000840302 |
| ENSG00000163545 | NUAK2 | 1.116770607 | 1.66531E-15 | 1.25212E-13 |
| ENSG00000171004 | HS6ST2 | 1.116216454 | 0.005650282 | 0.042796752 |
| ENSG00000141668 | CBLN2 | 1.112758914 | 0.000247788 | 0.003010568 |
| ENSG00000101188 | NTSR1 | 1.112366764 | 2.63034E-17 | 2.30478E-15 |
| ENSG00000241749 | RPSAP52 | 1.111984287 | 0.002759312 | 0.023628353 |
| ENSG00000267221 | C17orf113 | 1.111712338 | 0.002789192 | 0.023836066 |
| ENSG00000109320 | NFKB1 | 1.109922032 | 2.60227E-55 | 1.54812E-52 |
| ENSG00000166250 | CLMP | 1.108108195 | 3.68228E-09 | 1.29123E-07 |
| ENSG00000189068 | VSTM1 | 1.105750679 | 0.003518722 | 0.028960452 |
| ENSG00000169429 | CXCL8 | 1.104088628 | 1.78468E-10 | 7.70935E-09 |
| ENSG00000116691 | MIIP | 1.102746405 | 3.18662E-20 | 3.5429E-18 |
| ENSG00000171843 | MLLT3 | 1.101348405 | 0.00022345 | 0.00274536 |
| ENSG00000077150 | NFKB2 | 1.101018436 | 1.5987E-47 | 7.52941E-45 |
| ENSG00000149564 | ESAM | 1.100512141 | 4.74666E-06 | 9.18194E-05 |
| ENSG00000073670 | ADAM11 | 1.089556079 | 2.56195E-05 | 0.000404639 |
| ENSG00000135439 | AGAP2 | 1.086885958 | 0.001543541 | 0.014347994 |
| ENSG00000162078 | ZG16B | 1.080995922 | 2.85536E-05 | 0.000444765 |
| ENSG00000174791 | RIN1 | 1.078454669 | 5.33563E-17 | 4.512E-15 |
| ENSG00000178695 | KCTD12 | 1.075773984 | 2.31777E-13 | 1.43685E-11 |
| ENSG00000206341 | HLA-H | 1.075445788 | 1.43663E-07 | 3.85717E-06 |
| ENSG00000122862 | SRGN | 1.071456031 | 1.59007E-11 | 7.91768E-10 |
| ENSG00000150722 | PPP1R1C | 1.069916271 | 3.01591E-09 | 1.07088E-07 |
| ENSG00000259354 | AC025580.2 | 1.069767993 | 0.004282198 | 0.034047569 |
| ENSG00000198682 | PAPSS2 | 1.06456071 | 2.23709E-22 | 3.05886E-20 |
| ENSG00000167034 | NKX3-1 | 1.062826535 | 1.13982E-09 | 4.31377E-08 |
| ENSG00000140682 | TGFB1I1 | 1.059743282 | 5.63159E-11 | 2.60173E-09 |
| ENSG00000139192 | TAPBPL | 1.058720431 | 6.96643E-08 | 1.98681E-06 |
| ENSG00000161638 | ITGA5 | 1.057415515 | 1.12768E-10 | 5.01834E-09 |
| ENSG00000255248 | MIR100HG | 1.056830666 | 9.50989E-31 | 2.13563E-28 |
| ENSG00000147509 | RGS20 | 1.05134266 | 7.40306E-15 | 5.29615E-13 |
| ENSG00000117477 | CCDC181 | 1.047256716 | 2.67478E-05 | 0.000419722 |
| ENSG00000151474 | FRMD4A | 1.04623264 | 1.04964E-22 | 1.5082E-20 |
| ENSG00000136848 | DAB2IP | 1.046166805 | 9.49427E-11 | 4.24737E-09 |
| ENSG00000143127 | ITGA10 | 1.046062748 | 0.000345857 | 0.004006833 |
| ENSG00000150540 | HNMT | 1.04564423 | 0.003176165 | 0.026567281 |
| ENSG00000168394 | TAP1 | 1.040591737 | 4.86098E-30 | 1.04991E-27 |
| ENSG00000166311 | SMPD1 | 1.039026052 | 1.09896E-13 | 7.01801E-12 |
| ENSG00000012061 | ERCC1 | 1.038924437 | 1.51377E-21 | 1.92255E-19 |
| ENSG00000179981 | TSHZ1 | 1.038502156 | 5.01854E-22 | 6.64761E-20 |
| ENSG00000163364 | LINC01116 | 1.037543093 | 0.001363436 | 0.012860675 |
| ENSG00000171680 | PLEKHG5 | 1.032964767 | 9.72282E-18 | 8.81553E-16 |
| ENSG00000185716 | MOSMO | 1.032700004 | 2.65497E-52 | 1.42905E-49 |
| ENSG00000127252 | PLAAT1 | 1.02855268 | 3.45477E-10 | 1.42001E-08 |
| ENSG00000175183 | CSRP2 | 1.027463304 | 9.72404E-07 | 2.19828E-05 |
| ENSG00000096968 | JAK2 | 1.02714208 | 5.39434E-11 | 2.50693E-09 |
| ENSG00000112149 | CD83 | 1.025178321 | 1.51176E-16 | 1.22641E-14 |
| ENSG00000175344 | CHRNA7 | 1.025104556 | 0.005182733 | 0.03987894 |
| ENSG00000134070 | IRAK2 | 1.024307465 | 1.78321E-15 | 1.33485E-13 |
| ENSG00000124201 | ZNFX1 | 1.021031031 | 4.06918E-67 | 3.72935E-64 |
| ENSG00000117595 | IRF6 | 1.019353769 | 0.001394836 | 0.013116718 |
| ENSG00000104998 | IL27RA | 1.019319554 | 6.19278E-12 | 3.22576E-10 |
| ENSG00000243701 | DUBR | 1.016016906 | 1.17667E-05 | 0.000205675 |
| ENSG00000092929 | UNC13D | 1.01545529 | 0.000272144 | 0.003266536 |
| ENSG00000135749 | PCNX2 | 1.015283689 | 3.78179E-05 | 0.000569198 |
| ENSG00000164251 | F2RL1 | 1.01187586 | 9.60186E-39 | 3.19215E-36 |
| ENSG00000170166 | HOXD4 | 1.010155762 | 0.00242317 | 0.021232481 |
| ENSG00000086730 | LAT2 | 1.009728833 | 4.08655E-06 | 8.01475E-05 |
| ENSG00000171522 | PTGER4 | 1.00887521 | 1.16555E-24 | 1.8911E-22 |
| ENSG00000186472 | PCLO | 1.005535529 | 1.84771E-08 | 5.75353E-07 |
| ENSG00000204381 | LAYN | 1.004745919 | 1.58552E-08 | 5.02946E-07 |
| ENSG00000152377 | SPOCK1 | 1.003912107 | 5.56431E-06 | 0.000106063 |
| ENSG00000178445 | GLDC | 1.002859322 | 7.33553E-12 | 3.76891E-10 |
| ENSG00000114853 | ZBTB47 | 1.002327923 | 3.80109E-08 | 1.13464E-06 |
| ENSG00000274265 | AC245297.3 | 1.001338451 | 0.000208458 | 0.002582682 |
| ENSG00000182890 | GLUD2 | 1.001055695 | 0.00207333 | 0.018526122 |
