# Supplementary material for: Nuclear receptor coactivator 6 (NCoA6) promotes cell proliferation, migration, and invasion in pancreatic cancer
Source: Cancer Med. 2023 Aug 8;12(17):18425–39. doi: 10.1002/cam4.6427 (PMC10524018; doi:10.1002/cam4.6427)
Supplement: Supplementary file 2 — Table S2. [file CAM4-12-18425-s005.doc]

Supplementary Table 2. The DEGs that were significantly downregulated in the RNA-sequencing dataset of NCoA6-NC/sh PANC-1 cells.

| **Ensemble_ID** | **Symbol** | **log_2_FC** | ***P*value** | ***P*adj** |
| --- | --- | --- | --- | --- |
| ENSG00000163286 | ALPG | -7.277202934 | 1.5677E-71 | 1.56355E-68 |
| ENSG00000157404 | KIT | -6.772527329 | 6.24815E-65 | 5.29687E-62 |
| ENSG00000163283 | ALPP | -6.627578944 | 1.32587E-16 | 1.086E-14 |
| ENSG00000226239 | AL031658.1 | -6.260650369 | 3.13441E-14 | 2.11729E-12 |
| ENSG00000132329 | RAMP1 | -5.949803261 | 4.56865E-10 | 1.83124E-08 |
| ENSG00000156475 | PPP2R2B | -5.313231716 | 6.69877E-07 | 1.57419E-05 |
| ENSG00000113140 | SPARC | -5.232554095 | 8.00329E-82 | 9.79801E-79 |
| ENSG00000101331 | CCM2L | -5.154450695 | 1.14772E-27 | 2.17425E-25 |
| ENSG00000131771 | PPP1R1B | -5.108699415 | 3.98036E-14 | 2.66747E-12 |
| ENSG00000260903 | XKR7 | -5.096401036 | 7.83496E-58 | 5.1093E-55 |
| ENSG00000100302 | RASD2 | -4.793880695 | 4.21815E-75 | 4.61411E-72 |
| ENSG00000150556 | LYPD6B | -4.604296688 | 9.229E-06 | 0.000165848 |
| ENSG00000180875 | GREM2 | -4.603766761 | 3.81511E-05 | 0.000573196 |
| ENSG00000161905 | ALOX15 | -4.540078119 | 2.43908E-05 | 0.000387397 |
| ENSG00000272482 | AC254633.1 | -4.522965612 | 1.66954E-09 | 6.16041E-08 |
| ENSG00000172724 | CCL19 | -4.399639753 | 0.000290455 | 0.003449856 |
| ENSG00000279796 | AL133384.2 | -4.288316708 | 0.004675779 | 0.036643322 |
| ENSG00000095637 | SORBS1 | -4.282523494 | 2.51614E-32 | 5.92517E-30 |
| ENSG00000058335 | RASGRF1 | -4.27113948 | 8.33328E-07 | 1.91581E-05 |
| ENSG00000285636 | AL450163.1 | -4.008587756 | 0.004497467 | 0.035516792 |
| ENSG00000133636 | NTS | -4.002843424 | 6.60655E-05 | 0.000930736 |
| ENSG00000147255 | IGSF1 | -3.99560333 | 0.000690273 | 0.007244554 |
| ENSG00000187773 | DIPK1C | -3.958075719 | 0.00075071 | 0.007761148 |
| ENSG00000101198 | NKAIN4 | -3.880798142 | 6.95987E-17 | 5.8563E-15 |
| ENSG00000180730 | SHISA2 | -3.83391137 | 2.87092E-09 | 1.02154E-07 |
| ENSG00000171303 | KCNK3 | -3.778537096 | 1.6663E-24 | 2.66529E-22 |
| ENSG00000264717 | NPY4R2 | -3.778267874 | 0.005991093 | 0.044916643 |
| ENSG00000275620 | FLJ16779 | -3.772383687 | 3.19411E-05 | 0.000492105 |
| ENSG00000221340 | RNU6ATAC18P | -3.749324957 | 0.003109513 | 0.026093441 |
| ENSG00000154065 | ANKRD29 | -3.695583475 | 0.001178563 | 0.011382815 |
| ENSG00000039068 | CDH1 | -3.685164234 | 1.78962E-18 | 1.71915E-16 |
| ENSG00000129675 | ARHGEF6 | -3.619972975 | 6.30195E-84 | 8.54796E-81 |
| ENSG00000174407 | MIR1-1HG | -3.564674404 | 5.10911E-08 | 1.49096E-06 |
| ENSG00000151136 | BTBD11 | -3.546480826 | 2.41917E-05 | 0.000384955 |
| ENSG00000160963 | COL26A1 | -3.545009071 | 7.2332E-15 | 5.18558E-13 |
| ENSG00000103811 | CTSH | -3.489289375 | 6.56315E-56 | 4.04648E-53 |
| ENSG00000118257 | NRP2 | -3.454859519 | 4.21533E-86 | 5.95591E-83 |
| ENSG00000162009 | SSTR5 | -3.450470984 | 1.89911E-10 | 8.17245E-09 |
| ENSG00000187908 | DMBT1 | -3.340341029 | 0.005711148 | 0.043142134 |
| ENSG00000064205 | CCN5 | -3.262699318 | 2.3632E-97 | 4.71389E-94 |
| ENSG00000104413 | ESRP1 | -3.262128805 | 1.09886E-21 | 1.42223E-19 |
| ENSG00000198711 | SSBP3-AS1 | -3.261575273 | 0.002427216 | 0.021261592 |
| ENSG00000112139 | MDGA1 | -3.259823696 | 1.19355E-35 | 3.40111E-33 |
| ENSG00000204174 | NPY4R | -3.21402495 | 0.004176285 | 0.033402373 |
| ENSG00000149295 | DRD2 | -3.18960401 | 4.35986E-15 | 3.17942E-13 |
| ENSG00000081923 | ATP8B1 | -3.170598368 | 4.0828E-103 | 9.88924E-100 |
| ENSG00000168447 | SCNN1B | -3.167391764 | 8.08724E-14 | 5.22359E-12 |
| ENSG00000053747 | LAMA3 | -3.140247278 | 3.9992E-29 | 8.07219E-27 |
| ENSG00000173698 | ADGRG2 | -3.136305347 | 9.07862E-37 | 2.72439E-34 |
| ENSG00000230109 | LINC02643 | -3.104313346 | 2.52478E-05 | 0.000399325 |
| ENSG00000062038 | CDH3 | -3.098522754 | 1.33471E-40 | 4.81489E-38 |
| ENSG00000095303 | PTGS1 | -3.09344822 | 1.07045E-05 | 0.000190146 |
| ENSG00000198768 | APCDD1L | -3.07553872 | 1.81651E-06 | 3.85469E-05 |
| ENSG00000255141 | HNRNPA1P76 | -3.053276937 | 0.006440243 | 0.047631111 |
| ENSG00000169439 | SDC2 | -3.044483214 | 1.30687E-65 | 1.13631E-62 |
| ENSG00000041982 | TNC | -3.002805942 | 1.34304E-05 | 0.000230245 |
| ENSG00000198125 | MB | -2.99175039 | 5.93459E-19 | 5.88427E-17 |
| ENSG00000140545 | MFGE8 | -2.985148908 | 6.892E-135 | 2.1246E-131 |
| ENSG00000182870 | GALNT9 | -2.947713126 | 0.000382692 | 0.004367918 |
| ENSG00000174403 | MIR1-1HG-AS1 | -2.85250467 | 4.4703E-59 | 3.09363E-56 |
| ENSG00000124212 | PTGIS | -2.851898957 | 2.25938E-35 | 6.22892E-33 |
| ENSG00000171722 | SPATA46 | -2.835470168 | 0.000140088 | 0.001827775 |
| ENSG00000184845 | DRD1 | -2.808759787 | 2.38188E-10 | 1.00962E-08 |
| ENSG00000226900 | AL451069.1 | -2.790373187 | 1.06624E-06 | 2.38654E-05 |
| ENSG00000279118 | AC093535.2 | -2.770012386 | 3.99254E-05 | 0.000596945 |
| ENSG00000204175 | GPRIN2 | -2.742569413 | 6.19691E-26 | 1.08879E-23 |
| ENSG00000169783 | LINGO1 | -2.741004132 | 1.69539E-08 | 5.3331E-07 |
| ENSG00000111110 | PPM1H | -2.726114948 | 3.52492E-53 | 2.06086E-50 |
| ENSG00000069702 | TGFBR3 | -2.715957334 | 1.4913E-33 | 3.71839E-31 |
| ENSG00000197406 | DIO3 | -2.713940441 | 2.12865E-11 | 1.04461E-09 |
| ENSG00000235587 | GAPDHP65 | -2.701076185 | 0.000615697 | 0.006563438 |
| ENSG00000167676 | PLIN4 | -2.699599832 | 9.93445E-25 | 1.6196E-22 |
| ENSG00000088320 | REM1 | -2.691475623 | 0.002522425 | 0.021960314 |
| ENSG00000185924 | RTN4RL1 | -2.689782791 | 0.000153657 | 0.001975924 |
| ENSG00000204110 | LINC02520 | -2.655580871 | 0.001874387 | 0.016981157 |
| ENSG00000185885 | IFITM1 | -2.652477896 | 4.12091E-39 | 1.38357E-36 |
| ENSG00000166828 | SCNN1G | -2.646310209 | 4.34023E-13 | 2.59519E-11 |
| ENSG00000138207 | RBP4 | -2.616775292 | 3.71961E-16 | 2.9265E-14 |
| ENSG00000179869 | ABCA13 | -2.606972722 | 0.000441041 | 0.004945667 |
| ENSG00000198929 | NOS1AP | -2.60206136 | 1.50871E-25 | 2.58386E-23 |
| ENSG00000168350 | DEGS2 | -2.573131214 | 0.000304291 | 0.003587801 |
| ENSG00000162849 | KIF26B | -2.569624041 | 4.00768E-15 | 2.92889E-13 |
| ENSG00000124839 | RAB17 | -2.569489732 | 1.58871E-07 | 4.23864E-06 |
| ENSG00000174640 | C3orf36 | -2.565849829 | 3.58848E-08 | 1.07401E-06 |
| ENSG00000277494 | GPIHBP1 | -2.565838195 | 2.90919E-08 | 8.83176E-07 |
| ENSG00000201557 | SNORD114-15 | -2.564144222 | 0.001163167 | 0.011262987 |
| ENSG00000135643 | KCNMB4 | -2.55861122 | 6.4914E-10 | 2.54184E-08 |
| ENSG00000234928 | LINC01659 | -2.558275792 | 0.000868165 | 0.008814216 |
| ENSG00000188039 | NWD1 | -2.557931282 | 3.79574E-07 | 9.43647E-06 |
| ENSG00000163485 | ADORA1 | -2.554655852 | 3.65349E-05 | 0.000552586 |
| ENSG00000156510 | HKDC1 | -2.547075486 | 0.000948214 | 0.009479347 |
| ENSG00000187122 | SLIT1 | -2.543003675 | 3.97527E-06 | 7.81458E-05 |
| ENSG00000236078 | LINC01447 | -2.518546028 | 1.69891E-06 | 3.62556E-05 |
| ENSG00000166033 | HTRA1 | -2.507414069 | 9.62075E-07 | 2.17638E-05 |
| ENSG00000145908 | ZNF300 | -2.506561247 | 0.005394774 | 0.04125773 |
| ENSG00000204385 | SLC44A4 | -2.501371698 | 3.36015E-05 | 0.000513486 |
| ENSG00000275895 | U2AF1L5 | -2.49869942 | 0.000917882 | 0.009216871 |
| ENSG00000258498 | DIO3OS | -2.476318725 | 2.78913E-08 | 8.48246E-07 |
| ENSG00000107738 | VSIR | -2.466917533 | 2.477E-24 | 3.94343E-22 |
| ENSG00000239887 | C1orf226 | -2.463150741 | 1.02916E-17 | 9.30633E-16 |
| ENSG00000241362 | RPL36AP43 | -2.462003533 | 0.002623051 | 0.022707196 |
| ENSG00000112175 | BMP5 | -2.45080848 | 0.000122026 | 0.001617881 |
| ENSG00000164746 | C7orf57 | -2.449714765 | 3.67693E-09 | 1.29073E-07 |
| ENSG00000197561 | ELANE | -2.448923331 | 0.005497922 | 0.041923602 |
| ENSG00000134775 | FHOD3 | -2.427387955 | 1.41702E-22 | 2.0105E-20 |
| ENSG00000143850 | PLEKHA6 | -2.413202946 | 4.54583E-13 | 2.70437E-11 |
| ENSG00000251301 | LINC02384 | -2.410664596 | 1.72777E-08 | 5.41486E-07 |
| ENSG00000259207 | ITGB3 | -2.407129479 | 1.93429E-18 | 1.84766E-16 |
| ENSG00000166689 | PLEKHA7 | -2.402100266 | 9.76752E-44 | 3.99056E-41 |
| ENSG00000132334 | PTPRE | -2.389059592 | 3.89622E-05 | 0.000584088 |
| ENSG00000173080 | RXFP4 | -2.388093964 | 2.54546E-06 | 5.24401E-05 |
| ENSG00000117791 | MTARC2 | -2.385390518 | 8.68656E-22 | 1.12859E-19 |
| ENSG00000099194 | SCD | -2.382909566 | 2.20697E-20 | 2.51135E-18 |
| ENSG00000271447 | MMP28 | -2.377542217 | 0.002479489 | 0.021647651 |
| ENSG00000117115 | PADI2 | -2.363724542 | 3.66737E-06 | 7.28107E-05 |
| ENSG00000144596 | GRIP2 | -2.328201175 | 3.77911E-24 | 5.93285E-22 |
| ENSG00000130513 | GDF15 | -2.3165182 | 7.01473E-53 | 3.96449E-50 |
| ENSG00000285294 | LINC00842 | -2.312051534 | 0.003283739 | 0.02731214 |
| ENSG00000114698 | PLSCR4 | -2.310781932 | 4.46644E-21 | 5.38993E-19 |
| ENSG00000140511 | HAPLN3 | -2.298640177 | 5.69683E-87 | 8.3991E-84 |
| ENSG00000155761 | SPAG17 | -2.295962689 | 8.58514E-07 | 1.96704E-05 |
| ENSG00000196136 | SERPINA3 | -2.294450237 | 0.000626145 | 0.006664339 |
| ENSG00000109654 | TRIM2 | -2.290161183 | 4.27221E-33 | 1.04223E-30 |
| ENSG00000172346 | CSDC2 | -2.28644819 | 0.000829509 | 0.008466402 |
| ENSG00000227051 | C14orf132 | -2.282639712 | 5.49148E-32 | 1.27545E-29 |
| ENSG00000168542 | COL3A1 | -2.277358906 | 5.40184E-07 | 1.29636E-05 |
| ENSG00000013297 | CLDN11 | -2.27352679 | 6.49467E-05 | 0.000916496 |
| ENSG00000164741 | DLC1 | -2.266708565 | 1.10903E-32 | 2.64839E-30 |
| ENSG00000283283 | AC013268.4 | -2.2657543 | 0.004034627 | 0.032551556 |
| ENSG00000269873 | AC245884.10 | -2.255963233 | 0.004966594 | 0.038504161 |
| ENSG00000170421 | KRT8 | -2.252604093 | 2.00098E-19 | 2.09424E-17 |
| ENSG00000171840 | NINJ2 | -2.24783146 | 0.00257722 | 0.022385635 |
| ENSG00000288031 | AC093838.2 | -2.244573961 | 0.003419392 | 0.028267089 |
| ENSG00000233695 | GAS6-AS1 | -2.231053163 | 3.52224E-05 | 0.000535601 |
| ENSG00000001617 | SEMA3F | -2.230250423 | 1.57448E-28 | 3.10411E-26 |
| ENSG00000066468 | FGFR2 | -2.228137005 | 4.71311E-13 | 2.79898E-11 |
| ENSG00000276345 | LOC107987373 | -2.226044363 | 7.47262E-05 | 0.001036764 |
| ENSG00000233608 | TWIST2 | -2.22566142 | 4.56553E-07 | 1.11459E-05 |
| ENSG00000261713 | SSTR5-AS1 | -2.204173792 | 0.000267808 | 0.003222624 |
| ENSG00000162496 | DHRS3 | -2.201090781 | 1.6238E-141 | 5.5062E-138 |
| ENSG00000240032 | LNCSRLR | -2.196226144 | 2.52372E-05 | 0.000399325 |
| ENSG00000019186 | CYP24A1 | -2.193524039 | 0.004333634 | 0.034423405 |
| ENSG00000101096 | NFATC2 | -2.186705971 | 1.02439E-09 | 3.90303E-08 |
| ENSG00000138772 | ANXA3 | -2.167849996 | 2.04214E-05 | 0.000331335 |
| ENSG00000092969 | TGFB2 | -2.167659346 | 6.8868E-105 | 1.7964E-101 |
| ENSG00000256546 | LOC100506691 | -2.165303442 | 0.000911407 | 0.009162707 |
| ENSG00000198478 | SH3BGRL2 | -2.165112415 | 8.36637E-17 | 7.02237E-15 |
| ENSG00000207523 | SNORA66 | -2.161044039 | 0.002926161 | 0.024757018 |
| ENSG00000129514 | FOXA1 | -2.152329801 | 1.36113E-05 | 0.000232993 |
| ENSG00000115468 | EFHD1 | -2.151173335 | 8.05941E-19 | 7.92158E-17 |
| ENSG00000165091 | TMC1 | -2.132432027 | 0.002240215 | 0.019813692 |
| ENSG00000169129 | AFAP1L2 | -2.131518912 | 1.61062E-66 | 1.43726E-63 |
| ENSG00000185442 | FAM174B | -2.123965121 | 2.29001E-22 | 3.11865E-20 |
| ENSG00000287242 | AC073593.2 | -2.110990043 | 0.0041154 | 0.033045993 |
| ENSG00000165816 | VWA2 | -2.107366006 | 5.78698E-06 | 0.000109936 |
| ENSG00000137819 | PAQR5 | -2.104623414 | 7.84834E-06 | 0.000143239 |
| ENSG00000150275 | PCDH15 | -2.101332062 | 1.61933E-08 | 5.12234E-07 |
| ENSG00000111319 | SCNN1A | -2.10112591 | 5.25882E-06 | 0.000100693 |
| ENSG00000187848 | P2RX2 | -2.099544002 | 0.000648786 | 0.0068751 |
| ENSG00000163359 | COL6A3 | -2.094013531 | 1.21679E-10 | 5.39365E-09 |
| ENSG00000171596 | NMUR1 | -2.093561211 | 1.42972E-06 | 3.09196E-05 |
| ENSG00000064787 | BCAS1 | -2.07081922 | 3.17969E-12 | 1.72938E-10 |
| ENSG00000279145 | AC011912.1 | -2.067474201 | 0.000527277 | 0.005769587 |
| ENSG00000186188 | FFAR4 | -2.059430705 | 8.78358E-08 | 2.46974E-06 |
| ENSG00000165029 | ABCA1 | -2.049043115 | 1.34317E-07 | 3.63212E-06 |
| ENSG00000196415 | PRTN3 | -2.047781481 | 1.444E-05 | 0.000244586 |
| ENSG00000155324 | GRAMD2B | -2.047656536 | 6.98085E-28 | 1.32989E-25 |
| ENSG00000169435 | RASSF6 | -2.044473302 | 0.003056961 | 0.025703332 |
| ENSG00000131981 | LGALS3 | -2.03092409 | 1.09115E-37 | 3.39457E-35 |
| ENSG00000115596 | WNT6 | -2.029719808 | 0.005601076 | 0.042519029 |
| ENSG00000254285 | KRT8P3 | -2.029613248 | 2.77451E-15 | 2.0453E-13 |
| ENSG00000228484 | LOC101927692 | -2.023031427 | 0.000614005 | 0.006547451 |
| ENSG00000075461 | CACNG4 | -2.020411782 | 2.93543E-08 | 8.90345E-07 |
| ENSG00000186510 | CLCNKA | -2.017325853 | 0.003936899 | 0.031892083 |
| ENSG00000137198 | GMPR | -2.01688122 | 3.46623E-06 | 6.91817E-05 |
| ENSG00000151150 | ANK3 | -2.013664022 | 5.03242E-08 | 1.47112E-06 |
| ENSG00000254873 | AP001267.1 | -2.010843618 | 9.89061E-05 | 0.001338884 |
| ENSG00000132965 | ALOX5AP | -2.005034356 | 0.003326672 | 0.027594774 |
| ENSG00000184669 | OR7E14P | -2.002157924 | 0.002744157 | 0.02352234 |
| ENSG00000183114 | FAM43B | -1.997275742 | 0.000394202 | 0.004485705 |
| ENSG00000165424 | ZCCHC24 | -1.969514024 | 1.74154E-28 | 3.41362E-26 |
| ENSG00000170522 | ELOVL6 | -1.967644699 | 8.86857E-09 | 2.91408E-07 |
| ENSG00000176046 | NUPR1 | -1.962272133 | 1.58095E-16 | 1.27947E-14 |
| ENSG00000099204 | ABLIM1 | -1.939368692 | 8.32892E-35 | 2.22389E-32 |
| ENSG00000170153 | RNF150 | -1.933277934 | 0.000570634 | 0.006158096 |
| ENSG00000128849 | CGNL1 | -1.927571334 | 0.000306044 | 0.00360471 |
| ENSG00000277758 | LOC102724488 | -1.926404804 | 0.000599775 | 0.006417914 |
| ENSG00000184254 | ALDH1A3 | -1.923831508 | 5.34226E-23 | 8.01576E-21 |
| ENSG00000236510 | AC011284.1 | -1.921941339 | 0.003474661 | 0.028633232 |
| ENSG00000175274 | TP53I11 | -1.921202293 | 1.87157E-12 | 1.05423E-10 |
| ENSG00000254852 | NPIPA2 | -1.918649577 | 0.005460993 | 0.041698326 |
| ENSG00000286512 | AL031768.2 | -1.917461601 | 0.003524719 | 0.028996416 |
| ENSG00000101825 | MXRA5 | -1.915999492 | 6.85964E-06 | 0.000127249 |
| ENSG00000138131 | LOXL4 | -1.909628986 | 4.39297E-08 | 1.29761E-06 |
| ENSG00000124191 | TOX2 | -1.90344111 | 1.61863E-21 | 2.04806E-19 |
| ENSG00000138735 | PDE5A | -1.895477901 | 1.42095E-49 | 6.98326E-47 |
| ENSG00000148053 | NTRK2 | -1.89527622 | 2.82923E-16 | 2.24682E-14 |
| ENSG00000163083 | INHBB | -1.892328821 | 1.1594E-38 | 3.81701E-36 |
| ENSG00000167964 | RAB26 | -1.885796107 | 9.52685E-29 | 1.88921E-26 |
| ENSG00000170381 | SEMA3E | -1.857723439 | 2.45173E-19 | 2.54245E-17 |
| ENSG00000074527 | NTN4 | -1.850037523 | 4.84233E-20 | 5.31402E-18 |
| ENSG00000130203 | APOE | -1.844873495 | 7.56226E-23 | 1.10533E-20 |
| ENSG00000198939 | ZFP2 | -1.83240636 | 1.30771E-10 | 5.759E-09 |
| ENSG00000287750 | AL353572.4 | -1.829468513 | 0.002207356 | 0.019563888 |
| ENSG00000118898 | PPL | -1.820998969 | 2.11509E-08 | 6.51431E-07 |
| ENSG00000131242 | RAB11FIP4 | -1.814785145 | 4.20804E-19 | 4.24686E-17 |
| ENSG00000103460 | TOX3 | -1.813991953 | 0.000968696 | 0.009661315 |
| ENSG00000163624 | CDS1 | -1.81278152 | 2.1263E-06 | 4.4618E-05 |
| ENSG00000112293 | GPLD1 | -1.794989519 | 0.000534998 | 0.005833376 |
| ENSG00000223573 | TINCR | -1.791479515 | 6.68441E-08 | 1.91605E-06 |
| ENSG00000260807 | AC009041.2 | -1.782388245 | 1.32485E-05 | 0.000227703 |
| ENSG00000231187 | AL356056.2 | -1.775500142 | 0.003393264 | 0.028092183 |
| ENSG00000091536 | MYO15A | -1.772085831 | 0.001857239 | 0.016866352 |
| ENSG00000280399 | AC022497.1 | -1.772001049 | 0.000730483 | 0.007586726 |
| ENSG00000184347 | SLIT3 | -1.759845966 | 5.55112E-15 | 4.02219E-13 |
| ENSG00000260293 | AC106820.4 | -1.749172992 | 2.4125E-11 | 1.17203E-09 |
| ENSG00000115112 | TFCP2L1 | -1.747978214 | 1.54965E-12 | 8.80213E-11 |
| ENSG00000086619 | ERO1B | -1.747853321 | 5.20067E-17 | 4.40887E-15 |
| ENSG00000223044 | RNU6-130P | -1.745837813 | 0.00368847 | 0.030160606 |
| ENSG00000064195 | DLX3 | -1.743534835 | 1.30509E-10 | 5.75495E-09 |
| ENSG00000163435 | ELF3 | -1.738513542 | 1.4762E-15 | 1.11987E-13 |
| ENSG00000143320 | CRABP2 | -1.737298776 | 3.75257E-10 | 1.52761E-08 |
| ENSG00000204262 | COL5A2 | -1.72885528 | 2.70566E-09 | 9.67815E-08 |
| ENSG00000258988 | AL135978.1 | -1.724437494 | 0.000108124 | 0.001451778 |
| ENSG00000091136 | LAMB1 | -1.722292225 | 1.628E-07 | 4.32985E-06 |
| ENSG00000128510 | CPA4 | -1.719003343 | 0.002206629 | 0.01956256 |
| ENSG00000130876 | SLC7A10 | -1.706309405 | 0.000889095 | 0.008983675 |
| ENSG00000133424 | LARGE1 | -1.689102694 | 9.14981E-07 | 2.08375E-05 |
| ENSG00000116014 | KISS1R | -1.68445889 | 0.000107305 | 0.001442217 |
| ENSG00000265763 | ZNF488 | -1.675341891 | 2.49575E-10 | 1.05393E-08 |
| ENSG00000162733 | DDR2 | -1.674331273 | 4.14189E-11 | 1.96436E-09 |
| ENSG00000188580 | NKAIN2 | -1.672882582 | 7.81178E-06 | 0.000142725 |
| ENSG00000278456 | AC015712.7 | -1.671971215 | 0.001247314 | 0.011958275 |
| ENSG00000101197 | BIRC7 | -1.671856342 | 9.65295E-05 | 0.001310537 |
| ENSG00000128242 | GAL3ST1 | -1.661745524 | 0.003732526 | 0.030484097 |
| ENSG00000183971 | NPW | -1.661168528 | 1.14316E-06 | 2.53695E-05 |
| ENSG00000013588 | GPRC5A | -1.659982656 | 5.33807E-75 | 5.65669E-72 |
| ENSG00000165028 | NIPSNAP3B | -1.657665263 | 2.67937E-05 | 0.000420248 |
| ENSG00000109814 | UGDH | -1.656204133 | 1.09433E-58 | 7.42177E-56 |
| ENSG00000172717 | FAM71D | -1.65413248 | 2.93802E-13 | 1.80159E-11 |
| ENSG00000235169 | SMIM1 | -1.651889329 | 7.18283E-11 | 3.26501E-09 |
| ENSG00000135245 | HILPDA | -1.650778518 | 1.5431E-39 | 5.28549E-37 |
| ENSG00000166165 | CKB | -1.649219415 | 9.9662E-133 | 2.8163E-129 |
| ENSG00000171747 | LGALS4 | -1.642219343 | 0.00090311 | 0.009098175 |
| ENSG00000074416 | MGLL | -1.635447327 | 0.001103073 | 0.010754807 |
| ENSG00000108001 | EBF3 | -1.628293407 | 7.04718E-05 | 0.000983819 |
| ENSG00000035664 | DAPK2 | -1.625790271 | 3.18498E-05 | 0.000490921 |
| ENSG00000166446 | CDYL2 | -1.624140343 | 3.11909E-05 | 0.000482519 |
| ENSG00000134824 | FADS2 | -1.61627298 | 9.18433E-06 | 0.000165221 |
| ENSG00000234040 | RPL10P12 | -1.609837208 | 0.002984149 | 0.025166004 |
| ENSG00000260400 | AL513534.2 | -1.597282635 | 0.005143556 | 0.039680842 |
| ENSG00000103196 | CRISPLD2 | -1.596514566 | 4.14577E-10 | 1.6756E-08 |
| ENSG00000100292 | HMOX1 | -1.593218313 | 1.2325E-25 | 2.12152E-23 |
| ENSG00000285704 | AC004765.1 | -1.581153829 | 0.001331484 | 0.012626009 |
| ENSG00000124249 | KCNK15 | -1.575909654 | 1.41233E-07 | 3.80097E-06 |
| ENSG00000230615 | AL139220.2 | -1.57154711 | 3.57097E-05 | 0.000541313 |
| ENSG00000269028 | MTRNR2L12 | -1.570974033 | 0.000349873 | 0.004049208 |
| ENSG00000105641 | SLC5A5 | -1.564886711 | 1.54193E-05 | 0.000259102 |
| ENSG00000261051 | AC107021.2 | -1.560486034 | 1.24767E-05 | 0.000216302 |
| ENSG00000165731 | RET | -1.559679969 | 2.20589E-05 | 0.00035451 |
| ENSG00000170458 | CD14 | -1.558064568 | 0.000281469 | 0.003361959 |
| ENSG00000126562 | WNK4 | -1.557479072 | 0.000929746 | 0.009327721 |
| ENSG00000065534 | MYLK | -1.555501343 | 7.67179E-15 | 5.46534E-13 |
| ENSG00000073060 | SCARB1 | -1.554342097 | 4.05783E-38 | 1.31049E-35 |
| ENSG00000137193 | PIM1 | -1.54933108 | 2.87495E-23 | 4.39143E-21 |
| ENSG00000139044 | B4GALNT3 | -1.547287307 | 3.64628E-10 | 1.48791E-08 |
| ENSG00000223812 | AC073365.1 | -1.543572576 | 1.87237E-11 | 9.25541E-10 |
| ENSG00000108813 | DLX4 | -1.541650404 | 6.56913E-06 | 0.000122597 |
| ENSG00000243566 | UPK3B | -1.529587417 | 1.11552E-07 | 3.07538E-06 |
| ENSG00000060566 | CREB3L3 | -1.525390568 | 0.003140658 | 0.026322225 |
| ENSG00000005513 | SOX8 | -1.522671693 | 1.17408E-05 | 0.000205332 |
| ENSG00000152137 | HSPB8 | -1.522314262 | 0.001065296 | 0.010478521 |
| ENSG00000158715 | SLC45A3 | -1.518326914 | 4.11534E-13 | 2.46993E-11 |
| ENSG00000156453 | PCDH1 | -1.518020454 | 2.67182E-07 | 6.81726E-06 |
| ENSG00000204219 | TCEA3 | -1.513018951 | 2.02289E-10 | 8.68307E-09 |
| ENSG00000279662 | AC131649.2 | -1.511020258 | 5.1253E-07 | 1.237E-05 |
| ENSG00000008323 | PLEKHG6 | -1.510726664 | 3.94416E-15 | 2.88869E-13 |
| ENSG00000143867 | OSR1 | -1.506292587 | 0.002283432 | 0.020159123 |
| ENSG00000180720 | CHRM4 | -1.504770864 | 1.21429E-07 | 3.31268E-06 |
| ENSG00000079257 | LXN | -1.503280646 | 7.95797E-93 | 1.34927E-89 |
| ENSG00000183087 | GAS6 | -1.500694816 | 2.70349E-07 | 6.89289E-06 |
| ENSG00000134873 | CLDN10 | -1.49844633 | 2.36473E-09 | 8.53973E-08 |
| ENSG00000133056 | PIK3C2B | -1.494840552 | 8.85728E-25 | 1.45801E-22 |
| ENSG00000157570 | TSPAN18 | -1.490294895 | 0.001291476 | 0.012309248 |
| ENSG00000166025 | AMOTL1 | -1.487639876 | 6.11698E-51 | 3.24104E-48 |
| ENSG00000105649 | RAB3A | -1.476491588 | 0.003916319 | 0.031755714 |
| ENSG00000184012 | TMPRSS2 | -1.476039967 | 2.78765E-20 | 3.10951E-18 |
| ENSG00000162068 | NTN3 | -1.47429595 | 0.005682849 | 0.042985818 |
| ENSG00000078018 | MAP2 | -1.468716378 | 3.90751E-07 | 9.68594E-06 |
| ENSG00000228526 | MIR34AHG | -1.468221931 | 2.67326E-05 | 0.000419677 |
| ENSG00000207697 | MIR573 | -1.467063309 | 0.006414857 | 0.047495153 |
| ENSG00000135925 | WNT10A | -1.461288007 | 4.99441E-07 | 1.20799E-05 |
| ENSG00000105289 | TJP3 | -1.460671441 | 0.000386372 | 0.004406949 |
| ENSG00000160255 | ITGB2 | -1.458648635 | 4.19401E-05 | 0.00062213 |
| ENSG00000162576 | MXRA8 | -1.456535009 | 1.26673E-19 | 1.34655E-17 |
| ENSG00000203883 | SOX18 | -1.455793396 | 2.8554E-08 | 8.67622E-07 |
| ENSG00000139174 | PRICKLE1 | -1.452976386 | 0.001011308 | 0.010021461 |
| ENSG00000137834 | SMAD6 | -1.451968463 | 3.73495E-09 | 1.30569E-07 |
| ENSG00000184785 | SMIM10 | -1.449671713 | 6.26214E-22 | 8.26261E-20 |
| ENSG00000111321 | LTBR | -1.448571394 | 0.00072871 | 0.007572957 |
| ENSG00000227456 | LINC00310 | -1.443523721 | 0.005821263 | 0.043808042 |
| ENSG00000027075 | PRKCH | -1.441749537 | 0.000444862 | 0.00498028 |
| ENSG00000054277 | OPN3 | -1.441648706 | 1.58854E-12 | 9.00792E-11 |
| ENSG00000130635 | COL5A1 | -1.43967318 | 4.00369E-13 | 2.41146E-11 |
| ENSG00000182902 | SLC25A18 | -1.437172561 | 1.18021E-06 | 2.60382E-05 |
| ENSG00000077585 | GPR137B | -1.436769605 | 3.69402E-36 | 1.07986E-33 |
| ENSG00000169515 | CCDC8 | -1.433659111 | 2.81597E-10 | 1.17888E-08 |
| ENSG00000279039 | AC011447.6 | -1.428223386 | 0.002751988 | 0.02357159 |
| ENSG00000230333 | AC004160.1 | -1.426246624 | 0.002304958 | 0.020317422 |
| ENSG00000130822 | PNCK | -1.424770662 | 0.000790673 | 0.008127222 |
| ENSG00000158220 | ESYT3 | -1.424751519 | 0.000568125 | 0.006135386 |
| ENSG00000166145 | SPINT1 | -1.42267873 | 3.52904E-10 | 1.44879E-08 |
| ENSG00000226314 | ZNF192P1 | -1.422415878 | 0.003298619 | 0.027409008 |
| ENSG00000173482 | PTPRM | -1.421347601 | 0.00035023 | 0.004050578 |
| ENSG00000146555 | SDK1 | -1.420871886 | 0.002380965 | 0.020889659 |
| ENSG00000287262 | AC021148.2 | -1.418830037 | 0.000595394 | 0.006383123 |
| ENSG00000171345 | KRT19 | -1.418555109 | 8.6512E-78 | 9.77874E-75 |
| ENSG00000112182 | BACH2 | -1.417448427 | 0.00107193 | 0.010526832 |
| ENSG00000254815 | LMNTD2-AS1 | -1.41612066 | 8.81219E-06 | 0.000159456 |
| ENSG00000141232 | TOB1 | -1.4156291 | 2.41172E-46 | 1.0621E-43 |
| ENSG00000126895 | AVPR2 | -1.412249149 | 0.000163104 | 0.002080833 |
| ENSG00000164309 | CMYA5 | -1.411246106 | 0.00020221 | 0.002519077 |
| ENSG00000132561 | MATN2 | -1.409448778 | 0.000133162 | 0.001750207 |
| ENSG00000113594 | LIFR | -1.403938895 | 8.68691E-07 | 1.98901E-05 |
| ENSG00000185046 | ANKS1B | -1.403842053 | 0.002051958 | 0.018359336 |
| ENSG00000268756 | AC104534.1 | -1.398246257 | 1.50469E-07 | 4.02714E-06 |
| ENSG00000126500 | FLRT1 | -1.394970968 | 0.000318708 | 0.003738284 |
| ENSG00000272168 | CASC15 | -1.393447373 | 0.005157403 | 0.039729108 |
| ENSG00000169583 | CLIC3 | -1.390525506 | 4.67269E-05 | 0.000681746 |
| ENSG00000053254 | FOXN3 | -1.390213072 | 5.92791E-10 | 2.33197E-08 |
| ENSG00000286214 | AUXG01000058.1 | -1.389976615 | 8.00383E-09 | 2.6479E-07 |
| ENSG00000185985 | SLITRK2 | -1.388369777 | 2.06132E-09 | 7.49993E-08 |
| ENSG00000157303 | SUSD3 | -1.383871287 | 6.4714E-06 | 0.00012124 |
| ENSG00000111057 | KRT18 | -1.383819945 | 2.70481E-20 | 3.03709E-18 |
| ENSG00000164932 | CTHRC1 | -1.383743125 | 2.24275E-12 | 1.24471E-10 |
| ENSG00000273812 | BX640514.2 | -1.382066519 | 3.69977E-05 | 0.000558589 |
| ENSG00000119946 | CNNM1 | -1.381931773 | 2.31323E-30 | 5.02831E-28 |
| ENSG00000095587 | TLL2 | -1.381679555 | 0.001971619 | 0.01775766 |
| ENSG00000179528 | LBX2 | -1.380695181 | 3.0885E-07 | 7.76936E-06 |
| ENSG00000072310 | SREBF1 | -1.380495363 | 2.50829E-24 | 3.97458E-22 |
| ENSG00000124406 | ATP8A1 | -1.378647903 | 3.97321E-06 | 7.81458E-05 |
| ENSG00000066185 | ZMYND12 | -1.376904683 | 0.000395606 | 0.004498661 |
| ENSG00000151006 | PRSS53 | -1.372189507 | 6.88348E-06 | 0.000127551 |
| ENSG00000111186 | WNT5B | -1.37217973 | 4.38937E-06 | 8.55376E-05 |
| ENSG00000138944 | SHISAL1 | -1.372065529 | 0.004983014 | 0.038613807 |
| ENSG00000205744 | DENND1C | -1.371951108 | 0.00159308 | 0.014723722 |
| ENSG00000171992 | SYNPO | -1.371279874 | 5.54712E-14 | 3.63835E-12 |
| ENSG00000119888 | EPCAM | -1.367446336 | 5.79011E-24 | 9.00654E-22 |
| ENSG00000198646 | NCOA6 | -1.36523542 | 2.68776E-13 | 1.65412E-11 |
| ENSG00000257298 | AC008147.2 | -1.363460105 | 0.001171098 | 0.011322419 |
| ENSG00000175155 | YPEL2 | -1.363105027 | 0.001078235 | 0.010579553 |
| ENSG00000021826 | CPS1 | -1.361383784 | 1.02295E-06 | 2.29724E-05 |
| ENSG00000065320 | NTN1 | -1.355786781 | 0.000963452 | 0.009614672 |
| ENSG00000134802 | SLC43A3 | -1.352118831 | 0.00043707 | 0.004904386 |
| ENSG00000189184 | PCDH18 | -1.349268238 | 2.82408E-12 | 1.55287E-10 |
| ENSG00000143512 | HHIPL2 | -1.348263906 | 4.73559E-09 | 1.63195E-07 |
| ENSG00000163683 | SMIM14 | -1.344020126 | 3.91731E-12 | 2.10183E-10 |
| ENSG00000244242 | IFITM10 | -1.338145744 | 0.001156686 | 0.011207663 |
| ENSG00000126777 | KTN1 | -1.327687952 | 4.29307E-40 | 1.51644E-37 |
| ENSG00000185100 | ADSS1 | -1.324113496 | 5.45915E-07 | 1.30642E-05 |
| ENSG00000230650 | AC140479.2 | -1.320100834 | 2.2635E-08 | 6.95878E-07 |
| ENSG00000069812 | HES2 | -1.31865921 | 1.63715E-05 | 0.000272915 |
| ENSG00000135324 | MRAP2 | -1.30946957 | 0.001900485 | 0.017194625 |
| ENSG00000187017 | ESPN | -1.309265033 | 1.19157E-07 | 3.25856E-06 |
| ENSG00000221995 | TIAF1 | -1.305653232 | 1.50239E-09 | 5.56179E-08 |
| ENSG00000078114 | NEBL | -1.304513233 | 6.64632E-05 | 0.000934785 |
| ENSG00000050165 | DKK3 | -1.303930962 | 4.41856E-12 | 2.34848E-10 |
| ENSG00000244578 | LINC01391 | -1.302548207 | 0.001937736 | 0.01748965 |
| ENSG00000254682 | AP002387.2 | -1.302490018 | 5.27255E-05 | 0.00075888 |
| ENSG00000101144 | BMP7 | -1.301037549 | 0.001569327 | 0.014539858 |
| ENSG00000055118 | KCNH2 | -1.294662648 | 0.001421034 | 0.013322438 |
| ENSG00000065717 | TLE2 | -1.294628757 | 0.000405499 | 0.004597284 |
| ENSG00000251169 | LINC01843 | -1.291393562 | 0.000425553 | 0.004797376 |
| ENSG00000160999 | SH2B2 | -1.290988272 | 2.79918E-06 | 5.71808E-05 |
| ENSG00000162444 | RBP7 | -1.286108929 | 2.57349E-07 | 6.58122E-06 |
| ENSG00000213228 | RPL12P38 | -1.283890877 | 0.000554217 | 0.006006232 |
| ENSG00000092607 | TBX15 | -1.279675237 | 1.75654E-05 | 0.000290415 |
| ENSG00000188613 | NANOS1 | -1.275562506 | 1.90685E-08 | 5.90515E-07 |
| ENSG00000224094 | RPS24P8 | -1.274115794 | 5.40102E-16 | 4.22976E-14 |
| ENSG00000132026 | RTBDN | -1.26932761 | 4.52608E-05 | 0.000663838 |
| ENSG00000135312 | HTR1B | -1.268617907 | 2.90006E-10 | 1.20961E-08 |
| ENSG00000106571 | GLI3 | -1.267391156 | 0.006729211 | 0.049455473 |
| ENSG00000270504 | AL391422.4 | -1.264604063 | 4.32059E-07 | 1.05861E-05 |
| ENSG00000210195 | MT-TT | -1.264163974 | 0.005560595 | 0.042249555 |
| ENSG00000232913 | PLCE1-AS2 | -1.263656631 | 0.005747985 | 0.043372087 |
| ENSG00000182632 | CCNYL2 | -1.26178191 | 4.19887E-12 | 2.23522E-10 |
| ENSG00000043039 | BARX2 | -1.258921057 | 1.31411E-11 | 6.59028E-10 |
| ENSG00000125354 | SEPTIN6 | -1.255180806 | 2.48963E-11 | 1.20777E-09 |
| ENSG00000073711 | PPP2R3A | -1.253478666 | 1.07489E-16 | 8.89009E-15 |
| ENSG00000172985 | SH3RF3 | -1.249825278 | 0.001251223 | 0.011985583 |
| ENSG00000108984 | MAP2K6 | -1.247287079 | 5.22964E-05 | 0.000753664 |
| ENSG00000116977 | LGALS8 | -1.247184508 | 2.69865E-28 | 5.22922E-26 |
| ENSG00000148848 | ADAM12 | -1.246675629 | 0.001266904 | 0.012111843 |
| ENSG00000170558 | CDH2 | -1.243563904 | 6.17505E-47 | 2.86844E-44 |
| ENSG00000243943 | ZNF512 | -1.241460142 | 5.71315E-19 | 5.68132E-17 |
| ENSG00000099330 | OCEL1 | -1.238817633 | 1.43059E-14 | 9.98176E-13 |
| ENSG00000049130 | KITLG | -1.234524219 | 0.00043383 | 0.004874474 |
| ENSG00000085831 | TTC39A | -1.232708281 | 8.10285E-13 | 4.72922E-11 |
| ENSG00000179403 | VWA1 | -1.225761287 | 2.49082E-21 | 3.08261E-19 |
| ENSG00000269352 | PTOV1-AS2 | -1.224317318 | 0.00474914 | 0.03706406 |
| ENSG00000178531 | CTXN1 | -1.218154232 | 4.77631E-13 | 2.83155E-11 |
| ENSG00000250644 | AC068580.4 | -1.215529472 | 0.003388846 | 0.028062455 |
| ENSG00000166669 | ATF7IP2 | -1.214868223 | 4.31228E-33 | 1.0445E-30 |
| ENSG00000183346 | CABCOCO1 | -1.214735691 | 3.0255E-06 | 6.12141E-05 |
| ENSG00000253636 | AC022893.1 | -1.213569932 | 0.000889613 | 0.008986232 |
| ENSG00000157216 | SSBP3 | -1.212859065 | 2.48273E-06 | 5.121E-05 |
| ENSG00000203721 | LINC00862 | -1.209858239 | 0.001168238 | 0.011299189 |
| ENSG00000187098 | MITF | -1.203322734 | 0.002944448 | 0.024893104 |
| ENSG00000107902 | LHPP | -1.202556338 | 2.89873E-06 | 5.89305E-05 |
| ENSG00000116678 | LEPR | -1.202000512 | 0.000516028 | 0.005666611 |
| ENSG00000198947 | DMD | -1.195863174 | 1.94199E-07 | 5.09303E-06 |
| ENSG00000224546 | EIF4BP3 | -1.193236618 | 0.000660261 | 0.00698361 |
| ENSG00000287787 | AC092275.1 | -1.190991154 | 0.003785908 | 0.030830969 |
| ENSG00000165879 | FRAT1 | -1.187862688 | 1.29279E-10 | 5.70815E-09 |
| ENSG00000272808 | LOC105369201 | -1.178586499 | 0.000203568 | 0.002533206 |
| ENSG00000141293 | SKAP1 | -1.17416054 | 0.002909416 | 0.024646093 |
| ENSG00000168710 | AHCYL1 | -1.173421603 | 6.109E-14 | 3.99145E-12 |
| ENSG00000267280 | TBX2-AS1 | -1.172637025 | 0.00449908 | 0.035521261 |
| ENSG00000120278 | PLEKHG1 | -1.171894861 | 7.93317E-07 | 1.83878E-05 |
| ENSG00000255026 | AC136475.3 | -1.171343588 | 0.004479077 | 0.035404544 |
| ENSG00000006016 | CRLF1 | -1.170804371 | 4.08404E-05 | 0.000608478 |
| ENSG00000205277 | MUC12 | -1.170245245 | 0.000260556 | 0.003145406 |
| ENSG00000169894 | MUC3A | -1.168122668 | 1.09356E-08 | 3.54519E-07 |
| ENSG00000182912 | TSPEAR-AS2 | -1.166985537 | 1.01092E-08 | 3.293E-07 |
| ENSG00000100784 | RPS6KA5 | -1.166467582 | 1.70899E-05 | 0.000283799 |
| ENSG00000196839 | ADA | -1.161366674 | 1.89115E-35 | 5.34407E-33 |
| ENSG00000138439 | FAM117B | -1.160009291 | 1.69476E-09 | 6.23988E-08 |
| ENSG00000237813 | AC002066.1 | -1.159748788 | 0.00129953 | 0.012373087 |
| ENSG00000267892 | AC022144.1 | -1.15939386 | 0.001261772 | 0.01206618 |
| ENSG00000167191 | GPRC5B | -1.158691053 | 6.6692E-08 | 1.9133E-06 |
| ENSG00000162878 | PKDCC | -1.155883417 | 7.97066E-05 | 0.001099613 |
| ENSG00000251562 | MALAT1 | -1.153504594 | 6.8311E-22 | 8.9784E-20 |
| ENSG00000163590 | PPM1L | -1.148327772 | 7.15631E-35 | 1.94136E-32 |
| ENSG00000099875 | MKNK2 | -1.145557719 | 1.59639E-15 | 1.20565E-13 |
| ENSG00000235823 | OLMALINC | -1.145228433 | 7.83417E-06 | 0.000143057 |
| ENSG00000071575 | TRIB2 | -1.144870461 | 1.17128E-10 | 5.1987E-09 |
| ENSG00000196502 | SULT1A1 | -1.144785766 | 0.000569134 | 0.006144326 |
| ENSG00000263325 | AC003965.2 | -1.144080045 | 0.001308145 | 0.01244297 |
| ENSG00000235609 | AF127577.4 | -1.14309364 | 8.03266E-07 | 1.86057E-05 |
| ENSG00000256771 | ZNF253 | -1.142095109 | 1.02823E-09 | 3.91008E-08 |
| ENSG00000081059 | TCF7 | -1.139032752 | 1.12453E-36 | 3.34498E-34 |
| ENSG00000268658 | LINC00664 | -1.137132832 | 6.09869E-06 | 0.000114943 |
| ENSG00000112655 | PTK7 | -1.134878964 | 0.000352489 | 0.004066999 |
| ENSG00000152952 | PLOD2 | -1.132695642 | 3.32993E-14 | 2.24489E-12 |
| ENSG00000141401 | IMPA2 | -1.129445717 | 2.22318E-05 | 0.00035712 |
| ENSG00000205362 | MT1A | -1.124563146 | 1.07697E-06 | 2.4072E-05 |
| ENSG00000184497 | TMEM255B | -1.124239219 | 1.27109E-06 | 2.78261E-05 |
| ENSG00000131941 | RHPN2 | -1.121052989 | 2.74138E-18 | 2.58942E-16 |
| ENSG00000162804 | SNED1 | -1.11568787 | 2.81967E-09 | 1.00542E-07 |
| ENSG00000041353 | RAB27B | -1.114246361 | 0.0001357 | 0.001778736 |
| ENSG00000138336 | TET1 | -1.113887007 | 4.1125E-08 | 1.21688E-06 |
| ENSG00000099994 | SUSD2 | -1.112997255 | 6.22182E-08 | 1.79559E-06 |
| ENSG00000064309 | CDON | -1.111985296 | 2.57339E-05 | 0.000406255 |
| ENSG00000232677 | LINC00665 | -1.111558072 | 2.0142E-05 | 0.000327586 |
| ENSG00000198910 | L1CAM | -1.108627663 | 1.02854E-09 | 3.91008E-08 |
| ENSG00000253406 | AC012613.2 | -1.106565823 | 0.004932079 | 0.038262826 |
| ENSG00000187244 | BCAM | -1.105830283 | 2.02115E-05 | 0.000328243 |
| ENSG00000204386 | NEU1 | -1.100437326 | 1.66418E-12 | 9.4211E-11 |
| ENSG00000198155 | ZNF876P | -1.09947486 | 0.000859961 | 0.008742811 |
| ENSG00000227329 | AL139396.1 | -1.096337104 | 0.003749839 | 0.030603377 |
| ENSG00000106526 | ACTR3C | -1.095987908 | 1.32039E-05 | 0.000227166 |
| ENSG00000169239 | CA5B | -1.095434618 | 1.31948E-12 | 7.54527E-11 |
| ENSG00000177694 | NAALADL2 | -1.090423246 | 0.000130157 | 0.00171403 |
| ENSG00000185585 | OLFML2A | -1.090081585 | 1.30223E-05 | 0.000224497 |
| ENSG00000272079 | AC004233.2 | -1.083913416 | 0.002885345 | 0.024484995 |
| ENSG00000286760 | LOC100289495 | -1.083780711 | 0.000710992 | 0.007422954 |
| ENSG00000267481 | AC011477.2 | -1.082857707 | 0.006749562 | 0.04959429 |
| ENSG00000153246 | PLA2R1 | -1.079502635 | 9.96631E-05 | 0.00134692 |
| ENSG00000132688 | NES | -1.079313895 | 1.76969E-10 | 7.65434E-09 |
| ENSG00000143590 | EFNA3 | -1.077361795 | 1.10502E-05 | 0.00019557 |
| ENSG00000143162 | CREG1 | -1.075325747 | 2.57479E-06 | 5.298E-05 |
| ENSG00000102996 | MMP15 | -1.073066178 | 6.79418E-07 | 1.5944E-05 |
| ENSG00000101928 | MOSPD1 | -1.072969614 | 1.23239E-21 | 1.577E-19 |
| ENSG00000106031 | HOXA13 | -1.072646441 | 0.005983068 | 0.044876317 |
| ENSG00000105655 | ISYNA1 | -1.070467579 | 1.88891E-13 | 1.18179E-11 |
| ENSG00000178460 | MCMDC2 | -1.069466467 | 2.63157E-06 | 5.40499E-05 |
| ENSG00000139549 | DHH | -1.067245911 | 7.43047E-05 | 0.001031385 |
| ENSG00000073350 | LLGL2 | -1.062151231 | 1.27306E-17 | 1.13904E-15 |
| ENSG00000168209 | DDIT4 | -1.060264045 | 6.55518E-14 | 4.27042E-12 |
| ENSG00000235601 | BARX1-DT | -1.059272118 | 0.003069151 | 0.025793039 |
| ENSG00000180190 | TDRP | -1.055398495 | 0.000582097 | 0.006260362 |
| ENSG00000169710 | FASN | -1.052141684 | 2.51327E-19 | 2.59043E-17 |
| ENSG00000157021 | FAM92A1P1 | -1.051664617 | 0.000671072 | 0.007078083 |
| ENSG00000204839 | MROH6 | -1.049708634 | 6.13868E-07 | 1.45467E-05 |
| ENSG00000232940 | HCG25 | -1.048638934 | 0.000419908 | 0.004746361 |
| ENSG00000183508 | TENT5C | -1.047771791 | 1.60974E-09 | 5.94621E-08 |
| ENSG00000188171 | ZNF626 | -1.044717555 | 1.17785E-12 | 6.78116E-11 |
| ENSG00000185250 | PPIL6 | -1.04311426 | 0.003258461 | 0.02715364 |
| ENSG00000270025 | BMS1P7 | -1.040961172 | 0.003040054 | 0.025580207 |
| ENSG00000186197 | EDARADD | -1.040260142 | 1.61724E-14 | 1.12148E-12 |
| ENSG00000271789 | AL080317.1 | -1.038761599 | 0.005270967 | 0.040429428 |
| ENSG00000235027 | AC068580.3 | -1.038649495 | 0.005750118 | 0.043378529 |
| ENSG00000076555 | ACACB | -1.036143897 | 0.000635466 | 0.006752943 |
| ENSG00000025039 | RRAGD | -1.035806809 | 4.41415E-14 | 2.93498E-12 |
| ENSG00000104324 | CPQ | -1.029691996 | 1.17247E-13 | 7.47342E-12 |
| ENSG00000142910 | TINAGL1 | -1.029337664 | 0.000165109 | 0.002103673 |
| ENSG00000105991 | HOXA1 | -1.028883407 | 4.889E-08 | 1.4329E-06 |
| ENSG00000183049 | CAMK1D | -1.026242045 | 1.87309E-05 | 0.000307139 |
| ENSG00000160113 | NR2F6 | -1.021065881 | 2.46394E-22 | 3.32877E-20 |
| ENSG00000101098 | RIMS4 | -1.020170214 | 0.000224475 | 0.002755959 |
| ENSG00000210196 | MT-TP | -1.018513736 | 1.53277E-12 | 8.72082E-11 |
| ENSG00000157388 | CACNA1D | -1.01663717 | 1.59091E-20 | 1.82256E-18 |
| ENSG00000182759 | MAFA | -1.016623013 | 1.67058E-05 | 0.000278101 |
| ENSG00000203797 | DDO | -1.016543049 | 0.005796382 | 0.043640164 |
| ENSG00000121068 | TBX2 | -1.016457159 | 3.73843E-05 | 0.000563673 |
| ENSG00000088340 | FER1L4 | -1.013120547 | 1.22411E-20 | 1.41189E-18 |
| ENSG00000137225 | CAPN11 | -1.011841424 | 0.00239194 | 0.020980517 |
| ENSG00000104823 | ECH1 | -1.009599271 | 1.30972E-19 | 1.38789E-17 |
| ENSG00000259291 | ZNF710-AS1 | -1.006565496 | 1.76556E-07 | 4.67007E-06 |
| ENSG00000210082 | MT-RNR2 | -1.001889692 | 4.48858E-09 | 1.55156E-07 |
| ENSG00000130956 | HABP4 | -1.001634298 | 6.13333E-15 | 4.41574E-13 |
| ENSG00000232386 | AC015712.1 | -1.001042459 | 0.004630439 | 0.036355222 |
