# Supplementary material for: Nuclear receptor coactivator 6 (NCoA6) promotes cell proliferation, migration, and invasion in pancreatic cancer
Source: Cancer Med. 2023 Aug 8;12(17):18425–39. doi: 10.1002/cam4.6427 (PMC10524018; doi:10.1002/cam4.6427)
Supplement: Supplementary file 3 — Table S3. [file CAM4-12-18425-s006.doc]

Supplementary Table 3. The information of top 10 gene sets from GO BP analysis.

| **ID** | **Description** | **GeneRatio** | ***P*value** | ***P*adjust** | **Count** |
| --- | --- | --- | --- | --- | --- |
| GO:0009615 | response to virus | 55/909 | 7.74687E-14 | 3.06343E-10 | 55 |
| GO:0001819 | positive regulation of cytokine production | 63/909 | 1.62964E-13 | 3.06343E-10 | 63 |
| GO:0002237 | response to molecule of bacterial origin | 54/909 | 1.78513E-13 | 3.06343E-10 | 54 |
| GO:0030198 | extracellular matrix organization | 48/909 | 2.98156E-13 | 3.06343E-10 | 48 |
| GO:0043062 | extracellular structure organization | 48/909 | 3.38382E-13 | 3.06343E-10 | 48 |
| GO:0001667 | ameboidal-type cell migration | 63/909 | 3.5429E-13 | 3.06343E-10 | 63 |
| GO:0045229 | external encapsulating structure organization | 48/909 | 4.35036E-13 | 3.22424E-10 | 48 |
| GO:0001503 | ossification | 57/909 | 5.58604E-13 | 3.62255E-10 | 57 |
| GO:0051607 | defense response to virus | 44/909 | 7.08789E-13 | 3.67719E-10 | 44 |
| GO:0140546 | defense response to symbiont | 44/909 | 7.08789E-13 | 3.67719E-10 | 44 |
