# Supplementary material for: Nuclear receptor coactivator 6 (NCoA6) promotes cell proliferation, migration, and invasion in pancreatic cancer
Source: Cancer Med. 2023 Aug 8;12(17):18425–39. doi: 10.1002/cam4.6427 (PMC10524018; doi:10.1002/cam4.6427)
Supplement: Supplementary file 4 — Table S4. [file CAM4-12-18425-s002.doc]

Supplementary Table 4. The information of top 10 gene sets from GO CC analysis.

| **ID** | **Description** | **GeneRatio** | ***P*value** | ***P*adjust** | **Count** |
| --- | --- | --- | --- | --- | --- |
| GO:0062023 | collagen-containing extracellular matrix | 66/944 | 2.9139E-17 | 1.33165E-14 | 66 |
| GO:0009897 | external side of plasma membrane | 50/944 | 4.09401E-09 | 5.73698E-07 | 50 |
| GO:0045121 | membrane raft | 43/944 | 5.02143E-09 | 5.73698E-07 | 43 |
| GO:0098857 | membrane microdomain | 43/944 | 5.02143E-09 | 5.73698E-07 | 43 |
| GO:0005911 | cell-cell junction | 54/944 | 1.81262E-08 | 1.65674E-06 | 54 |
| GO:0030139 | endocytic vesicle | 40/944 | 1.37864E-07 | 1.05006E-05 | 40 |
| GO:0030666 | endocytic vesicle membrane | 27/944 | 6.57335E-07 | 3.40784E-05 | 27 |
| GO:0044853 | plasma membrane raft | 20/944 | 6.69131E-07 | 3.40784E-05 | 20 |
| GO:0005604 | basement membrane | 18/944 | 6.71128E-07 | 3.40784E-05 | 18 |
| GO:0005788 | endoplasmic reticulum lumen | 36/944 | 1.32136E-06 | 6.03863E-05 | 36 |
