# Supplementary material for: Nuclear receptor coactivator 6 (NCoA6) promotes cell proliferation, migration, and invasion in pancreatic cancer
Source: Cancer Med. 2023 Aug 8;12(17):18425–39. doi: 10.1002/cam4.6427 (PMC10524018; doi:10.1002/cam4.6427)
Supplement: Supplementary file 5 — Table S5. [file CAM4-12-18425-s004.doc]

Supplementary Table 5. The information of top 10 gene sets from GO MF analysis.

| **ID** | **Description** | **GeneRatio** | ***P*value** | ***P*adjust** | **Count** |
| --- | --- | --- | --- | --- | --- |
| GO:0005125 | cytokine activity | 41/918 | 2.06689E-12 | 1.65763E-09 | 41 |
| GO:0048018 | receptor ligand activity | 63/918 | 3.79321E-12 | 1.65763E-09 | 63 |
| GO:0030546 | signaling receptor activator activity | 63/918 | 7.75234E-12 | 2.25852E-09 | 63 |
| GO:0005539 | glycosaminoglycan binding | 39/918 | 1.77415E-11 | 3.87651E-09 | 39 |
| GO:0008201 | heparin binding | 32/918 | 3.77701E-11 | 6.60221E-09 | 32 |
| GO:1901681 | sulfur compound binding | 39/918 | 1.33365E-09 | 1.94268E-07 | 39 |
| GO:0005201 | extracellular matrix structural constituent | 30/918 | 1.91584E-09 | 2.39207E-07 | 30 |
| GO:0005126 | cytokine receptor binding | 34/918 | 7.53125E-07 | 8.22789E-05 | 34 |
| GO:0019955 | cytokine binding | 22/918 | 1.51094E-06 | 0.000146729 | 22 |
| GO:0070851 | growth factor receptor binding | 22/918 | 1.93174E-06 | 0.000168834 | 22 |
