# Supplementary material for: Nuclear receptor coactivator 6 (NCoA6) promotes cell proliferation, migration, and invasion in pancreatic cancer
Source: Cancer Med. 2023 Aug 8;12(17):18425–39. doi: 10.1002/cam4.6427 (PMC10524018; doi:10.1002/cam4.6427)
Supplement: Supplementary file 6 — Table S6. [file CAM4-12-18425-s003.doc]

Supplementary Table 6. The information of top 20 gene sets from KEGG analysis.

| **ID** | **Description** | **GeneRatio** | ***P*value** | ***P*adjust** | **Count** |
| --- | --- | --- | --- | --- | --- |
| hsa04668 | TNF signaling pathway | 28/488 | 5.2889E-11 | 1.59725E-08 | 28 |
| hsa04060 | Cytokine-cytokine receptor interaction | 43/488 | 4.20594E-08 | 6.35098E-06 | 43 |
| hsa04010 | MAPK signaling pathway | 42/488 | 1.0886E-07 | 1.09586E-05 | 42 |
| hsa05165 | Human papillomavirus infection | 43/488 | 1.11872E-06 | 8.44633E-05 | 43 |
| hsa04151 | PI3K-Akt signaling pathway | 44/488 | 2.79538E-06 | 0.000147641 | 44 |
| hsa04064 | NF-kappa B signaling pathway | 20/488 | 2.93326E-06 | 0.000147641 | 20 |
| hsa05202 | Transcriptional misregulation in cancer | 29/488 | 3.50867E-06 | 0.000151374 | 29 |
| hsa04514 | Cell adhesion molecules | 24/488 | 8.04357E-06 | 0.000303645 | 24 |
| hsa05412 | Arrhythmogenic right ventricular cardiomyopathy | 16/488 | 1.03071E-05 | 0.00034586 | 16 |
| hsa05134 | Legionellosis | 13/488 | 2.44784E-05 | 0.000739247 | 13 |
| hsa04390 | Hippo signaling pathway | 23/488 | 5.89326E-05 | 0.001617969 | 23 |
| hsa04640 | Hematopoietic cell lineage | 17/488 | 7.41522E-05 | 0.001866164 | 17 |
| hsa05146 | Amoebiasis | 17/488 | 0.000108988 | 0.002531875 | 17 |
| hsa05323 | Rheumatoid arthritis | 16/488 | 0.00011741 | 0.002532691 | 16 |
| hsa04657 | IL-17 signaling pathway | 16/488 | 0.000133816 | 0.002694166 | 16 |
| hsa05140 | Leishmaniasis | 14/488 | 0.000169586 | 0.00320094 | 14 |
| hsa04380 | Osteoclast differentiation | 19/488 | 0.0002148 | 0.003701909 | 19 |
| hsa05164 | Influenza A | 23/488 | 0.000220644 | 0.003701909 | 23 |
| hsa05410 | Hypertrophic cardiomyopathy | 15/488 | 0.000273932 | 0.004241228 | 15 |
| hsa04061 | Viral protein interaction with cytokine and cytokine receptor | 16/488 | 0.000280876 | 0.004241228 | 16 |
