# Supplementary material for: Nuclear receptor coactivator 6 (NCoA6) promotes cell proliferation, migration, and invasion in pancreatic cancer
Source: Cancer Med. 2023 Aug 8;12(17):18425–39. doi: 10.1002/cam4.6427 (PMC10524018; doi:10.1002/cam4.6427)
Supplement: Supplementary file 7 — Table S7. [file CAM4-12-18425-s001.doc]

Supplementary Table 7. The statistical values of SENESE_HDAC1_TARGETS_UP gene set from GSEA analysis.

| Description | Set Size | Enrichment Score | NES | *P*value | *P*adjust | qvalue |
| --- | --- | --- | --- | --- | --- | --- |
| SENESE_HDAC1_TARGETS_UP | 435 | 0.422525095 | 1.794021154 | 1.09389E-09 | 1.27013E-07 | 1.07393E-07 |
